# Conditioning and pseudoconditioning differently change intrinsic excitability of inhibitory interneurons in the neocortex

**DOI:** 10.1093/cercor/bhae109

**Published:** 2024-04-03

**Authors:** Dominik Kanigowski, Joanna Urban-Ciecko

**Affiliations:** Laboratory of Electrophysiology, Nencki Institute of Experimental Biology PAS, 3 Pasteur Street, 02-093 Warsaw, Poland; Laboratory of Electrophysiology, Nencki Institute of Experimental Biology PAS, 3 Pasteur Street, 02-093 Warsaw, Poland

**Keywords:** barrel cortex, in vitro electrophysiology, parvalbumin interneurons, somatostatin interneurons, VIP interneurons

## Abstract

Many studies indicate a broad role of various classes of GABAergic interneurons in the processes related to learning. However, little is known about how the learning process affects intrinsic excitability of specific classes of interneurons in the neocortex. To determine this, we employed a simple model of conditional learning in mice where vibrissae stimulation was used as a conditioned stimulus and a tail shock as an unconditioned one. In vitro whole-cell patch-clamp recordings showed an increase in intrinsic excitability of low-threshold spiking somatostatin-expressing interneurons (SST-INs) in layer 4 (L4) of the somatosensory (barrel) cortex after the conditioning paradigm. In contrast, pseudoconditioning reduced intrinsic excitability of SST-LTS, parvalbumin-expressing interneurons (PV-INs), and vasoactive intestinal polypeptide-expressing interneurons (VIP-INs) with accommodating pattern in L4 of the barrel cortex. In general, increased intrinsic excitability was accompanied by narrowing of action potentials (APs), whereas decreased intrinsic excitability coincided with AP broadening. Altogether, these results show that both conditioning and pseudoconditioning lead to plastic changes in intrinsic excitability of GABAergic interneurons in a cell-specific manner. In this way, changes in intrinsic excitability can be perceived as a common mechanism of learning-induced plasticity in the GABAergic system.

## Introduction

The activity of GABAergic interneurons is essential in the process of learning at the stage of the acquisition of new skills, as well as during expression of already acquired skills (Donato et al. 2013; Chen et al. 2015; Lipina et al. 2016; Adler et al. 2019; Krabbe et al. 2019; Cummings and Clem 2020). Moreover, the GABAergic interneuron function is also important for memory maintenance when a new learning acquisition process takes place (Adler et al. 2019). Experiments using multiple learning models have shown that GABAergic interneurons are actively involved in different types of learning and the formation of many types of memory (Wolff et al. 2014; Abbas et al. 2018; Khan et al. 2018; Adler et al. 2019; Turi et al. 2019; Xu et al. 2019; Melzer et al. 2021; Morales et al. 2021). Despite the growing knowledge of the participation of GABAergic interneurons in various brain areas in learning and memory, little is known about plastic changes of these cells in these processes.

Intrinsic excitability is the ability of a neuron to generate an action potential (AP) in response to an input signal and results from a unique composition of ion channels responsible for AP generation (Frick and Johnston 2005; Chen et al. 2020). Intrinsic excitability together with the number and strength of synaptic inputs constitutes overall neuronal excitability.

Neuronal intrinsic excitability can be subject to plastic changes as a result of different forms of learning or after exposure to an enriched environment (Malik and Chattarji 2012; McKay et al. 2013; Song et al. 2015). It has also been shown that neuronal excitability changes after other forms of experience, such as sensory deprivation (Breton and Stuart 2009), addiction, and exposure to stress factors (Kourrich and Thomas 2009; Francis et al. 2015; Rau et al. 2015). Moreover, pathological conditions such as epilepsy, ischemia, and other forms of damage to the nervous system affect neuronal excitability (Fan et al. 2008; Paz et al. 2010; Kirchheim et al. 2013).

Plastic changes in neuronal activity after learning might occur as a result of synaptic plasticity (Donato et al. 2013; Lucas et al. 2016) and/or as an effect of changes in intrinsic excitability (McKay et al. 2013; Cummings et al. 2022; Ferranti et al. 2022). Learning-related changes in intrinsic excitability of excitatory neurons have been observed after several forms of learning in multiple brain areas (Kuo et al. 2008; Bekisz et al. 2010; Motanis et al. 2014; Sehgal et al. 2014; Soler-Cedeño et al. 2016; Whitaker et al. 2017; Dunn et al. 2018). However, much less attention has been paid to studying learning-evoked changes in intrinsic excitability of several classes of GABAergic interneurons. This has resulted in a serious gap in the knowledge regarding the overall changes in the neuronal network after learning. Despite this, it is known that intrinsic excitability of many classes of GABAergic interneurons like somatostatin- (SST-INs), parvalbumin- (PV-INs), and vasoactive intestinal polypeptide-expressing interneurons (VIP-INs) may change in response to various factors like aging, inflammation, mild traumatic brain injury, or drugs application (Campanac and Hoffman 2013; Francavilla et al. 2020; Feng et al. 2021; Harris et al. 2022; Muchhala et al. 2022). Studies also suggest that intrinsic excitability of SST-, PV-, and VIP-INs may be related to the ability to learn and remember (McKay et al. 2013; Yi et al. 2014; Favuzzi et al. 2017; Yu et al. 2019; Francavilla et al. 2020; Monaco et al. 2020; Gould et al. 2021; Melzer et al. 2021; Armenta-Resendiz et al. 2022). However, it is unknown whether learning induces changes in intrinsic excitability of all molecularly distinct subtypes of neocortical GABAergic interneurons. To address this question, we subjected mice to simple forms of learning based on conditioning or pseudoconditioning paradigms consisting of a tactile stimulation of whiskers paired with an electric tail shock. Subsequently, we performed whole-cell patch-clamp recordings in acute brain slices to assess changes in intrinsic excitability of SST-, PV-, and VIP-INs located in layer 4 (L4) of the cortical representations of stimulated whiskers in the barrel cortex. We found that conditioning increases intrinsic excitability of low-threshold spiking SST-INs (SST-LTS), whereas pseudoconditioning decreases intrinsic excitability of SST-LTS, PV-INs, and accommodating VIP-INs (VIP-AC).

Our experiments demonstrate that intrinsic excitability of the 3 main molecularly distinct subtypes of neocortical GABAergic interneurons in L4 of the barrel cortex undergoes plastic changes after conditioning or pseudoconditioning. This indicates that changes in intrinsic excitability can be perceived as a common mechanism of learning-induced plasticity in the GABAergic circuit. Furthermore, these plastic changes are specific in terms of a cell type and a form of learning.

## Materials and methods

### Experimental animals

The experiments involved the progeny of animals of homozygous lines imported from The Jackson Laboratory (United States): SST-Cre (line number: 013044), PV-Cre (012358), VIP-Cre (010908), and Ai14 (007908). Double transgenic animals were obtained from the crossing of SST-Cre, PV-Cre, and VIP-Cre with the Ai14 line to obtain tdTomato expression following Cre-mediated recombination.

Mice were housed in the Animal Facility at the Nencki Institute of Experimental Biology PAS (Warsaw, Poland). Experiments were performed on P21-50 mice of both sexes. Animals with unlimited access to water and food were kept at a temperature of 20 to 23 °C and relative air humidity of 40% to 50%. The day in which the animals lived consisted of alternating 12 h cycles of light and dark phases. All experimental procedures were done in accordance with Directive 2010/63/EU of the European Parliament and the Council of 2010 September 22 on the protection of animals used for scientific purposes. All procedures were approved by the first Local Ethical Committee for Animal Experiments in Warsaw (permission numbers: 172/2016 and 841/2019). All efforts were made to minimize the number of animals used and their suffering.

### Learning paradigms

#### Habituation

Before the learning procedures, mice were habituated to the immobilization of the head and neck in a special immobilization device. A single habituation session to immobilization lasted 10 min a day for 5 consecutive days starting at P21. Habituation and subsequent learning sessions were performed at a fixed time in the first 2 h of the light phase.

#### Conditioned group (CS + UCS)

The day after the last habituation session, animals were subjected to aversive conditioning (Siucinska and Kossut 1996). During this procedure, the mouse was immobilized as in the habituation session, and the animal’s tail was connected with a clip to the ACS100 electrical stimulator (Circlelabs, Poland). Then, using a brush, a row B of vibrissae on the left side of the mouse’s snout was stroked manually from the back of the snout to its front, a conditioned stimulus (CS). Each stroke lasted for 3 s and was repeated 3 times. In the final second of the last stroke, an electric shock (0.5 mA, 0.5 s) as the unconditioned stimulus (UCS) was applied to the mouse’s tail. After a 6 s interval, the trial was repeated. CS + UCS pairing was repeated 40 times, resulting in a 10 min session. Each animal in the CS + UCS group underwent 3 sessions on 3 consecutive days and received a total of 120 CS + UCS associations.

#### Pseudoconditioned group

In the Pseudoconditioned (Pseudo) group, the CS (stroking the vibrissae) was administered just like in the CS + UCS animals. However, the UCS (electric shock to the tail) was delivered in a randomized manner (not associated with the CS stimulus). Each mouse in the Pseudo group underwent the same number of sessions as the CS + UCS group.

#### Naïve group

The animals of the Naïve (Naïve) group did not experience any manipulations by the experimenter.

#### Behavioral assessment

A part of the CS + UCS or Pseudo mice were filmed with a video camera during the course of training to assess behavioral outcomes of training (Cybulska-Klosowicz et al. 2009; Dobrzanski et al. 2022). The number of trials during which a mouse moved its head in response to stimulation of vibrissae was counted. Only trials during which an animal moved its head during the CS application were counted; head movements during both UCS application and intertrial intervals were not taken into account.

### Electrophysiological experiments

#### Brain sectioning

Approximately 24 h after the third session of CS + UCS or Pseudo, mice were subjected to inhalational anesthesia using isoflurane (~5% in inhaled air, Iso-Vet) for brain preparation. Only the right hemisphere was used for further experiments, also in the case of the Naïve group. Then, using a metal matrix, the frontal part of the brain was cut off at an angle of 45° to the sagittal plane, and the hemisphere was glued with its front side to the metal base of the vibratome (Leica VT1000 S, Germany). This mounting procedure ensures that the brain is sliced across 5 rows of barrels (A to E) to obtain sections containing one barrel from each row (Finnerty et al. 1999). The thickness of the slices was 350 μm. Sections were cut in a cooled (0 to 2 °C) artificial cerebrospinal fluid (ACSF) composed of (in mM): 113 NaCl, 2.5 KCl, 2 MgSO_4_, 2 CaCl_2_, 1 NaH_2_PO_4_, 26.2 NaHCO_3_, and 11 glucose, equilibrated with carbogen in a volume ratio of 95% O_2_/5% CO_2_. The slices were placed in a recovery chamber filled with carbogen-equilibrated ACSF solution and maintained at a constant temperature of 30 °C. After 5 min, the chamber with the slices was moved to room temperature for further recovery period.

#### Whole-cell patch-clam recordings

An individual slice was transferred to the recording chamber mounted under a Zeiss microscope. Barrels were visualized at 4× magnification, and slices with 5 barrels were taken for further analysis. Neurons were visualized at 40× magnification using infrared differential interference contrast and fluorescence. Whole-cell patch-clamp recordings were performed from fluorescently labeled interneurons located in L4 of the B barrel, which was the representation of one of the whiskers stimulated during learning procedures. For recordings, ACSF solution was composed of (in mM): 113 NaCl, 3.5 KCl, 0.5 MgSO_4_, 1 CaCl_2_, 1 NaH_2_PO_4_, 26.2 NaHCO_3_, and 11 glucose, equilibrated with carbogen 95% O_2_/5% CO_2_. Patch electrodes (5 to 8 MΩ) were filled with the internal solution composed of (in mM): 125 K-gluconate, 10 HEPES, 0.5 EGTA, 2 KCl, 4 ATP-Mg, 0.3 GTP-Na. Recordings were carried out at room temperature. Electrophysiological data were acquired using Clampex 10.6.2.2. software, a Multiclamp 700B amplifier, and an Axon Digidata 1550B analog-to-digital converter (Molecular Devices, United States). The analog signal was filtered at 3 kHz and sampled at 20 kHz.

The resting potential, access resistance, and input resistance were monitored online. Resistance parameters were measured in the current-clamp mode as a membrane response to a −10 pA pulse of 100 ms duration or in the voltage-clamp mode in response to a +10 mV pulse (10 to 50 ms). Recordings in which input or access resistance changes exceeded 30% of the initial value were excluded from further analysis.

Intrinsic excitability measurements were performed in the current-clamp mode at a −65 mV potential across the membrane. The neuronal membrane was depolarized using rectangular current pulses with a duration of 500 ms and an amplitude of the current intensity increasing in steps of 5 to 20 pA every 12 s. Depending on the interneuron type, the current intensity was increased by steps of 5 pA (VIP-INs), 10 pA (SST-INs), or 20 pA (PV-INs) until the depolarization block was observed after inducing the maximum frequency of discharges.

### Electrophysiological data analysis

Recorded cells were clustered into different electrophysiological subtypes based on firing patterns, according to the classification proposed by the Petilla group and others (Cauli et al. 1997; Ma et al. 2006; Ascoli et al. 2008; Prönneke et al. 2015).

Data from individual cells were used to plot the frequency–current curve (F–I). Then, the sigmoidal curve was fitted to this data using the nonlinear regression method in the SigmaPlot 14.0 program (Systat Software Inc., United States). The analysis of sigmoidal curves, as opposed to simple comparisons of *F*–*I* curves, allows for a more precise investigation of the dynamic in neuronal excitability. The sigmoidal curve was described by the function *f* = a*x^*b*/(*c*^*b* + *x*^*b*). In this way, intrinsic excitability of each cell was represented by the sigmoidal curve described by 3 parameters. The parameter “a” (curve’s maximum) corresponds to the maximum value reached by the sigmoidal curve and reflects the maximal firing frequency of a cell. The parameter “b” (curve’s steepness) determines the slope of the sigmoid curve; the higher “b” means the steeper of the middle part of the curve. This parameter has a dimensionless value and reflects the gain—the responsiveness of the neuronal spiking frequency to changes in the intensity of inputs (Bryson et al. 2020). The parameter “c” (curve’s midpoint) describes the midpoint of the sigmoidal curve, which is the point on the *x*-axis that corresponds to a half of the maximum value reached by the curve. This parameter defines the intensity of the injected current at which a cell discharges with a half of the maximal frequency. Only one cell from the group of VIP-INs in the pseudoconditioned mice was excluded from the analysis because the *F*–*I* curve was poorly fitted to the model (adjusted *R*^2^ ≥ 0.96).

Spike adaptation was analyzed at the maximum firing frequency and expressed as the adaptation index—the ratio of the last interspike interval to the first interval in the series of discharges.

The AP threshold was established by measuring a 5 mV change in the AP amplitude within a 1 ms interval. The basal potential was considered 1 ms before the threshold. The basal potential was used to measure AP parameters such as the AP amplitude, the half-width, and the fast afterhyperpolarization (AHP) amplitude. The AP amplitude was measured between the basal potential and the peak of the AP. The AP half-width was calculated as the duration at a half of the AP amplitude. The amplitude of the fast AHP was calculated between the point of the lowest potential within 2 ms after the peak of the AP and the basal membrane potential. The second AP in a series of discharges was analyzed because the first AP often significantly differs from the subsequent ones (Cauli et al. 1997; Deans et al. 2001; Beierlein et al. 2003). All the parameters of the AP shape were analyzed at the maximum firing frequency.

### Statistical analysis

Statistical analyses were performed with GraphPad Prism 8.0.2 (GraphPad Software, United States). Normality of the distribution was tested using the D’Agostino–Pearson test and equal variance was analyzed with Bartlett’s test. Parametric tests were used when data sets were distributed normally and tested groups did not differ in variances. Otherwise, nonparametric tests were employed. Data were analyzed using one-way analysis of variance (ANOVA) followed by Tukey’s post hoc test, Kruskal–Wallis test followed by Dunn’s post hoc test, unpaired *t*-test, Mann–Whitney test, Kolmogorov–Smirnov test with Bonferroni correction, or two-sample *Χ*^2^ test for equality of proportions with continuity correction. Unless otherwise stated, the results are presented as the mean ± SEM and were considered statistically significant when *P* < 0.05. Values exceeding 3 times the sample standard deviation were excluded from further analysis. No more than 10% of samples were removed from further analysis in a single test. The figure legends indicate the number of neurons and animals in the following scheme: number of cells (number of mice).

## Results

### Conditioning increases intrinsic excitability of L4 SST-LTS

To visualize SST-INs, we used double heterozygous offspring of SST-Cre and Ai14 mice, hereinafter referred to as SST-Ai14. In this mouse line, SST-INs express a red fluorescent marker (tdTomato), which enables successful discrimination of these interneurons from other cells.

**Fig. 1:**
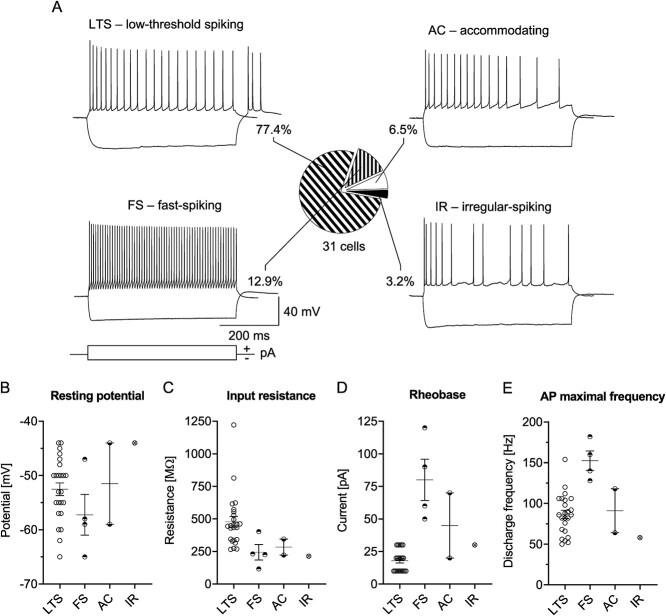
Electrophysiological subtypes of L4 SST-INs in the barrel cortex of Naïve mice. A) Example traces of SST-IN spiking responses and the pie chart showing the distribution of different spiking patterns. B-E) Basic electrophysiological parameters of SST-INs with four different spiking phenotypes. A) Naïve = 31 (16). B-E) LTS = 24 (12), FS = 4 (4), AC = 2 (2), IR = 1 (1).

In the first step, we analyzed L4 SST-INs of the Naïve group of mice. We distinguished four electrophysiological subpopulations of these interneurons based on membrane properties, spiking patterns, the presence of rebound spikes, and the maximal frequency of spiking (Fig. 1A–E). Out of 31 recorded SST-INs in 16 Naïve animals, 24 neurons were classified as LTS and possessed rebound spikes (77.4% of recorded cells), 4 neurons were fast-spiking (FS, 12.9%), 2 neurons were accommodating (AC, 6.5%), and 1 cell displayed an irregular firing (IR, 3.2%). All these firing phenotypes were previously observed in cortical SST-INs (Wang et al. 2004; Ma et al. 2006; Nigro et al. 2018). Further analysis was carried out on L4 SST-INs with the LTS pattern (SST-LTS) because they constituted the dominant class of SST-INs.

To verify whether simple forms of learning in mice induce plastic changes in intrinsic excitability of SST-INs, mice were subjected to 2 different paradigms of learning, conditioning or pseudoconditioning. SST-Ai14 mice were randomly assigned to one of the 3 groups: Naïve, CS + UCS, and Pseudo. The learning paradigm was done based on the whisker-to-barrel cortex system according to the previous protocol (Siucinska and Kossut 1996). Learning-induced behavioral changes were assessed as a reduction in head movements (minifreezing) toward the CS stimulus in the course of the training (Cybulska-Klosowicz et al. 2009). Conditioning reduced the percentage of head movements toward the CS stimulus, indicating that learning occurred in this group of mice (Fig. S1–S15). Also, pseudoconditioned mice showed a reduction of the percentage of head movements toward CS stimulus; however, the degree of minifreezing on the third day of the training was lower in the Pseudo group compared to CS + UCS mice (Fig. S1B and C).

One day after the last session of CS + UCS or Pseudo, animals were sacrificed to obtain acute brain slices for whole-cell patch-clamp recordings. All recordings were done from SST-INs located in L4 of the barrel cortex in barrels corresponding to the manipulated row of whiskers.

First, we analyzed the distribution of electrophysiological subtypes of L4 SST-INs among 3 groups of mice (Fig. S2) to answer the question of whether the learning protocol changes the firing phenotypes. This analysis did not reveal significant differences in fractions of cells presenting different spiking patterns between the CS + UCS or Pseudo group relative to the Naïve, indicating that learning protocols did not change firing phenotypes of L4 SST-INs.

The analysis of SST-LTS basic electrophysiological parameters showed no differences in resting membrane potential, input resistance, or rheobase between the tested groups of mice (Fig. S3). Earlier studies using the same learning model have shown an increase in intrinsic excitability of excitatory neurons located in the cortical representations of manipulated whiskers in CS + UCS animals (Bekisz et al. 2010).

**Fig. 2:**
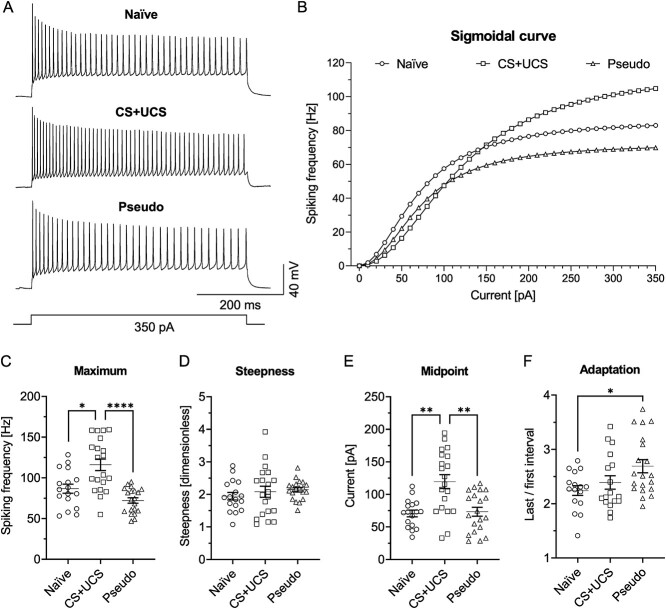
Conditioning increases intrinsic excitability of SST-LTS in L4 of the barrel cortex. A) Examples of cell discharges and B) averaged sigmoidal curves from three groups of mice tested. Both cell discharges and sigmoidal curves reached higher frequencies of APs in the CS + UCS group in comparison to the Naïve and Pseudo groups of mice. C) The curve’s maximum was higher in the CS + UCS group in relation to the Naïve (Kruskal-Wallis test, *P* ≤ 0.0001; Dunn's test, *P* = 0.0272) and to the Pseudo groups (Dunn's test, *P* ≤ 0.0001). D) There were no differences in the steepness of the curves between the groups (Kruskal-Wallis test, *P* = 0.4258). E) The midpoint was higher in the CS + UCS group compared to the Naïve (Kruskal-Wallis test, *P* = 0.0011; Dunn's test, *P* = 0.0028) and the Pseudo groups (Dunn's test, *P* = 0.0071). F) The discharge adaptation was greater in the Pseudo group than in the Naïve group (One-way ANOVA, F_(2, 50)_ = 4.002, *P* = 0.0244; Tukey's test, *P* = 0.0225). B-E) Naïve = 17 (7), CS + UCS = 20 (10), Pseudo = 20 (6). F) Naïve = 16 (6), CS + UCS = 17 (10), Pseudo = 20 (6).

To investigate whether learning also causes plastic changes in cortical SST-INs, intrinsic excitability of SST-LTS of CS + UCS, Pseudo, and Naïve groups of mice were compared. To better assess the potential changes in intrinsic excitability of SST-LTS, the sigmoidal function fitting method was utilized (Fig. 2).

The sigmoidal curve was fitted to the *F*–*I* curve of every recorded cell. Thus, the excitability of an individual interneuron was described by the parameters of the sigmoidal curve. The parameter “a” (reflecting the maximum frequency of cell firing in response to depolarizing current stimulus) was higher in the CS + UCS group compared to the Naïve and Pseudo groups (Fig. 2A–C). The next 2 parameters, “b” and “c,” describe the dynamic of intrinsic excitability. The parameter “b” characterizes the slope of the curve in its middle; the larger the parameter is, the more S-shaped the curve becomes. The slope reflects the neuronal gain (Bryson et al. 2020). The analysis of the slope of the sigmoidal curves showed no significant differences between groups of animals (Fig. 2D). The parameter “c” indicates the current value at which cells respond with half of the maximum firing frequency, which corresponds to the midpoint of the curve. The values of the parameter “c” were higher in the CS + UCS group in relation to the Naïve and Pseudo groups (Fig. 2E).

We also analyzed raw data and mean *F*–*I* curves (Fig. S4). This analysis also confirmed higher intrinsic excitability of L4 SST-LTS in the CS + UCS group compared to the Naïve and Pseudo groups. Unexpectedly, the raw data analysis also revealed lower intrinsic excitability in the Pseudo group relative to the Naïve and CS + UCS groups, differences that were not shown by the sigmoidal curve analysis.

In the next step, an adaptation of a discharge frequency was tested (Ha and Cheong 2017). The adaptation of the firing frequency is often associated with learning and acquiring new memory traces (Reuveni and Barkai 2018). Moreover, the strength of spike adaptation affects the frequency as well as the neuronal firing pattern. Changes in spike adaptation and frequency have a significant impact on the synchronization and filtering of the input signal (Ermentrout et al. 2001; Gutkin et al. 2005; Ha et al. 2016). Here, the adaptation index reached higher values in L4 SST-LTS in the Pseudo group compared to the Naïve mice but not to the CS + UCS animals (Fig. 2F). The increased adaptation of neuronal discharges in the Pseudo group indicates a decrease in intrinsic excitability of SST-LTS in this group of animals, which is in line with *F*–*I* curve analysis (Fig. S4).

Summarizing, analysis of the sigmoid parameters and raw *F*–*I* curves indicates that intrinsic excitability of L4 SST-LTS is higher in CS + UCS mice compared to the Naïve and Pseudo animals. Moreover, the raw *F*–*I* curve analysis indicates that pseudoconditioning results in lower intrinsic excitability of L4 SST-LTS compared to the Naïve and CS + UCS animals.

Our further analysis focused on the effect of learning procedures on the AP parameters. This analysis was designed to assess what changes in the AP parameters may be associated with the increase in intrinsic excitability of SST-LTS after CS + UCS. We found that there were no differences in AP threshold potential between groups of mice (Fig. S5A and B). However, AP amplitude was lower in the CS + UCS than in the Naïve group (Fig. S5A and C) and AP half-width was narrower in the CS + UCS group compared to the Naïve and Pseudo (Fig. S5A and D). There were no differences in amplitude of AHPs between the groups (Fig. S5A and E).

Summarizing, our results indicate that conditioning in mice leads to an increase in intrinsic excitability of SST-LTS in the L4 of the barrel cortex in the representation of vibrissae stimulated during the learning procedure. The analysis of an AP shape suggests that the increase in the excitability of these interneurons after conditioning may result from the narrowing of the AP half-width. Inversely, pseudoconditioning causes a decrease in intrinsic excitability of L4 SST-LTS, which was not accompanied by changes in AP shape.

### Pseudoconditioning decreases intrinsic excitability of L4 PV-INs

To study whether simple forms of learning in mice induce plastic changes in intrinsic excitability of PV-INs, the PV-Ai14 mice were used to visualize PV-INs in slices.

**Fig. 3:**
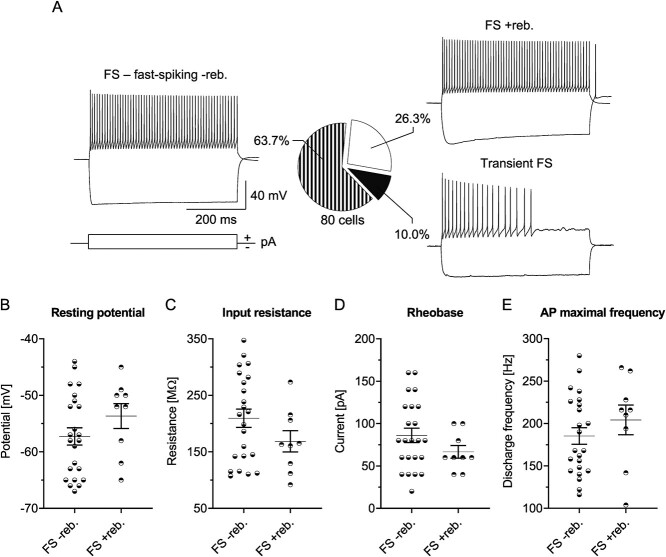
Electrophysiological subtypes of L4 PV-INs in the barrel cortex of Naïve mice. A) Example traces and the pie chart of PV-IN spiking patterns. B-E) Basic electrophysiological parameters of PV-INs with FS firing without (FS -reb.) and with (FS +reb.) rebound spikes. No differences were observed in B) resting potential (*P* = 0.2078); C) input resistance (*P* = 0.1623); D) rheobase (*P* = 0.1881); E) maximal frequency of APs (*P* = 0.3275). A) Naïve = 80 (27). B-E) Unpaired t-test; FS reb. = 23 (10), FS +reb. = 9 (7).

As in the case of SST-INs, we first analyzed basic electrophysiological properties and firing patterns of L4 PV-INs in the Naïve mice. Here, we found that PV-INs respond with the classical FS phenotype (Kawaguchi 1993; Kawaguchi 1995; Cauli et al. 1997). However, our in-depth observation suggested that the FS firing pattern might be divided into 3 subtypes (Fig. 3A).

Out of 80 PV-INs, 63.7% exhibited FS pattern without the rebound spikes (FS − reb.) and 26.3% displayed FS phenotype with rebound spikes (FS + reb.). A small subset of PV-INs (10.0%) responded with a transient FS pattern characterized by the cessation of discharges before the end of the depolarizing current step. This firing pattern was also previously observed in PV-INs (Kawaguchi 1993). The analysis of the basic membrane properties did not show any statistically significant differences in resting potential, input resistance, rheobase, or the maximal firing frequency between FS − reb. and FS + reb. (Fig. 3B–E). We also did not observe any differences in the sigmoidal curve parameters describing intrinsic excitability dynamics between cells with these two firing patterns (data not shown). These results prompted us to pool data obtained from both classes of FS PV-INs for further analysis. PV-INs with the transient FS firing were excluded from further analysis because the firing frequency in these interneurons was much lower than in the classical FS (data not shown).

The analysis of the distribution of electrophysiological subtypes of L4 PV-INs between groups of mice revealed a lower fraction of FS − reb. in the CS + UCS group compared to the Naïve group (Fig. S6). In turn, we observed an increase in the percentage of FS + reb. in CS + UCS relative to the Naïve group. The fraction of transient FS did not differ between groups. Changes in the percentage of FS + reb. and FS − reb. Between the Naïve and CS + UCS mice indicate that conditioning may influence activity and/or number of channels responsible for the generation of rebound spikes, presumably T-type and HCN channels (Kim et al. 2001; Molineux et al. 2006; Ascoli et al. 2010; Engbers et al. 2011). For further analysis, we pooled FS + reb. and FS − reb. into one group because we did not observe any differences between FS + reb. and FS − reb. regarding effects of learning paradigms (data not shown).

Next, we compared the basic electrophysiological properties of L4 PV-INs between animals that experienced different learning procedures. This analysis did not reveal any changes in resting potential nor rheobase of PV-INs between 3 groups of mice (Fig. S7A and C). However, we observed lower input resistance in the Pseudo mice than in the Naïve animals but not in the CS + UCS (Fig. S7B).

**Fig. 4:**
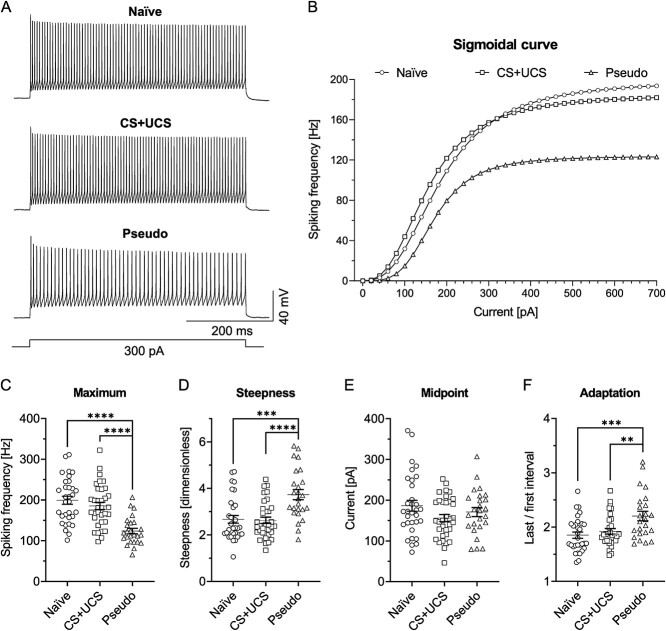
Pseudoconditioning decreases intrinsic excitability of PV-INs in L4 of the barrel cortex. A) Cell discharges and B) averaged sigmoidal curves from three groups of mice tested. The cell discharges and the sigmoidal curves present reduced frequencies of APs in the Pseudo group of mice in relation to the Naïve and CS + UCS groups. C) The curve’s maximum was lower in the Pseudo group in comparison to the Naïve (Kruskal-Wallis test, *P* < 0.0001; Dunn’s test, *P* < 0.0001) and CS + UCS groups of animals (Dunn’s test, *P* < 0.0001). D) The curve’s steepness was higher in Pseudo mice in relation to the Naïve (One-way ANOVA, F_(2, 88)_ = 12.24, *P* < 0.0001; Tukey’s test, *P* = 0.0001) and CS + UCS groups (Tukey’s test, *P* < 0.0001). E) No change in the curve’s midpoint was found between groups (One-way ANOVA, F_(2, 88)_ = 1.960, *P* = 0.1470). F) The discharge adaptation in the Pseudo group was higher in relation to the Naïve (One-way ANOVA, F_(2, 84)_ = 7.769, *P* = 0.0008; Tukey’s test, *P* = 0.0008) and CS + UCS groups (Tukey’s test, *P* = 0.0088). B-E) Naïve = 32 (14), CS + UCS = 34 (11), Pseudo = 25 (10). F) Naïve = 16 (6), CS + UCS = 17 (10), Pseudo = 20 (6).

The subsequent statistical comparison of the maximal values (the parameter “a”) of the sigmoidal curves revealed that PV-INs in the Pseudo group exhibited a significantly lower maximum discharge frequency compared to the Naïve and CS + UCS groups (Fig. 4A–C).

The parameter “b” characterizing the slope of sigmoidal curves reached much higher values in the Pseudo group than in the Naïve and CS + UCS mice, indicating lower gain in intrinsic excitability of L4 PV-INs after pseudoconditioning (Fig. 4D). The parameter “c” defining the midpoint of the curve was similar in all groups (Fig. 4E). In addition, the comparison of the adaptation ratio showed greater spike adaptation in the Pseudo group compared to the Naïve and CS + UCS groups (Fig. 4F).

We also performed analysis on raw data and *F*–*I* curves, which mirrored results obtained by the sigmoidal curve analysis, further confirming the decrease in intrinsic excitability of L4 PV-INs following pseudoconditioning (Fig. S8).

Altogether, these results suggest that pseudoconditioning causes a decrease in L4 PV-IN intrinsic excitability. The reduction in intrinsic excitability was manifested by a decrease in the maximal firing frequency and the gain, as well as by stronger spike adaptation.

To shed light on the mechanisms of the reduction in intrinsic excitability of L4 PV-INs in pseudoconditioned mice, the shapes of APs were analyzed, as was done for the SST-LTS studies. The analysis of the AP shape parameters showed that the threshold potential in PV-INs had a significantly hyperpolarized value in the CS + UCS group relative to the Naïve and Pseudo groups (Fig. S9A and B). There were no differences between the groups of mice in terms of AP amplitude (Fig. S9A and C). However, the AP half-width was significantly wider in the Pseudo group than in the Naïve and CS + UCS mice (Fig. S9A and D). Finally, the mean amplitude of fast AHPs was larger in the Pseudo group compared to the Naïve and CS + UCS groups of animals (Fig. S9A and E). In summary, lower input resistance, wider APs, and deeper AHPs might be responsible for decreased intrinsic excitability of PV-INs after pseudoconditioning.

### Pseudoconditioning decreases intrinsic excitability of L4 VIP-AC

**Fig. 5:**
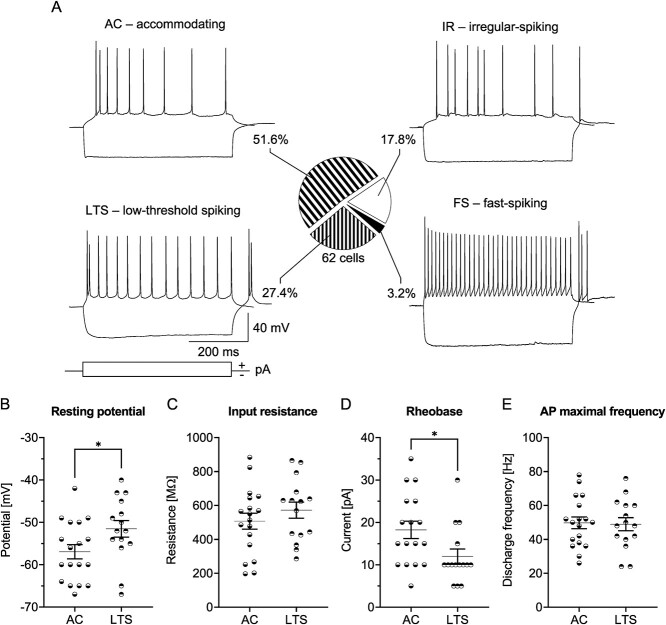
Electrophysiological subtypes of L4 VIP-INs in the barrel cortex of Naïve mice. A) Example traces and the pie chart of four electrophysiological subtypes of VIP-INs in L4 of the barrel cortex. B-E) The comparison of basic electrophysiological properties between VIP-INs presenting AC or LTS spiking pattern. B) The resting potential was depolarized in VIP-LTS in comparison to VIP-AC (*P* = 0.0425). C) The input resistance was similar between the firing subtypes (*P* = 0.3405). D) The rheobase was higher in VIP-AC than VIP-LTS (*P* = 0.0204). E) There were no differences in the maximal frequency of APs between the two subtypes of interneurons (*P* = 0.8714). A) Naïve = 62 (31). B, C, E) Unpaired t-test; AC = 18 (13), LTS = 15 (10). D) Mann-Whitney test; AC = 17 (12), LTS = 15 (10).

To fully understand how learning influences intrinsic excitability of 3 main classes of cortical GABAergic interneurons, we also analyzed L4 VIP-INs. The analysis of VIP-IN spiking patterns in response to depolarizing current steps revealed four firing subtypes (Fig. 5).

Out of 62 VIP-INs in 31 Naïve mice, 32 (51.6%) neurons exhibited AC pattern, 17 (27.4%) cells were LTS, 11 (17.8%) IR, and 2 (3.2%) FS (Fig. 5A). The AC and IR patterns of VIP-INs were previously observed in the rodent cerebral cortex, but LTS and FS did not (Caputi et al. 2009; Prönneke et al. 2015; He et al. 2016; Schuman et al. 2019). However, other researchers can consider the LTS in our classification as a type of AC pattern.

Due to the diversity of the VIP-IN population in terms of spiking patterns, only the 2 most numerous types were selected for further analysis: AC and LTS. First, both firing subtypes of VIP-INs were compared within the data obtained from Naïve mice. We found that VIP-INs with AC spiking pattern (VIP-AC) had more hyperpolarized resting potential and higher rheobase than VIP-INs with LTS pattern (VIP-LTS) (Fig. 5B and D). Both cell subtypes presented, however, similar values of input resistance and AP maximal frequency (Fig. 5C and E). Due to the differences in the resting potential and rheobase between VIP-AC and VIP-LTS in control animals, the analysis of the impact of learning on VIP-IN intrinsic excitability was performed separately for these 2 firing subtypes.

The analysis of the distribution of VIP-IN firing patterns across tested mice did not reveal any changes in firing phenotypes after learning procedures (Fig. S10).

**Fig. 6:**
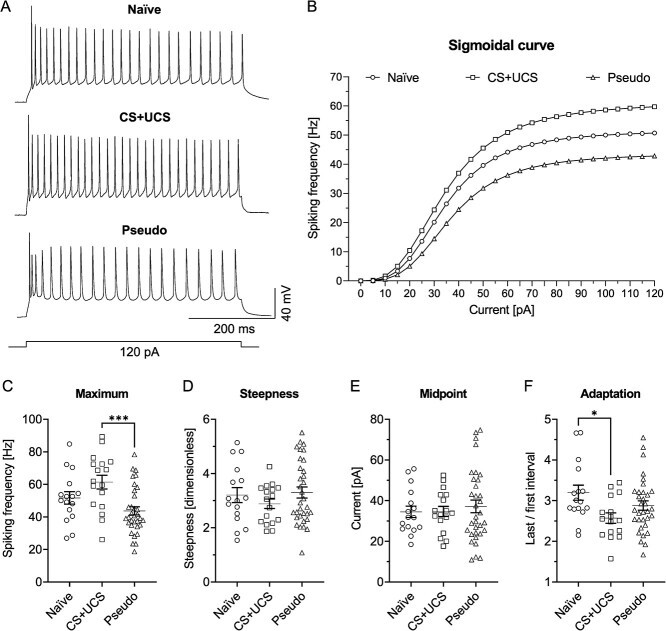
Intrinsic excitability of VIP-AC differs between conditioned and pseudoconditioned groups of mice. A, B) The CS + UCS and Pseudo groups vary in the AP frequency, as shown by A) the examples of firing responses as well as by B) averaged sigmoidal curves. C) The maximal frequency of APs was decreased in the Pseudo group in contrast to the CS + UCS mice (One-way ANOVA, F_(2, 63)_ = 7.431, *P* = 0.0013; Tukey’s test, *P* = 0.0009), but not the Naïve mice. D) The curve’s steepness was comparable between groups (One-way ANOVA, F_(2, 63)_ = 0.8808, *P* = 0.4195). E) The curve’s midpoint did not differ between groups (Kruskal-Wallis test, *P* = 0.9813). F) The spike adaptation was lower in the CS + UCS group relative to the Naïve group (One-way ANOVA, F_(2, 62)_ = 3.670, *P* = 0.0312; Tukey’s test, *P* = 0.0234). B-E) Naïve = 16 (11), CS + UCS = 17 (10), Pseudo = 33 (20). F) Naïve = 16 (11), CS + UCS = 16 (10), Pseudo = 33 (20).

To verify how conditioning and pseudoconditioning influence VIP-AC, we first compared the basic electrophysiological properties of these interneurons between the Naïve, CS + UCS, and Pseudo groups of animals. This analysis revealed more depolarized values of resting potential in the Pseudo group in relation to the Naïve and CS + UCS mice (Fig. S11A). However, no differences were observed between groups in terms of input resistance and rheobase (Fig. S11B and C). Next, we analyzed intrinsic excitability of VIP-AC following learning procedures (Fig. 6).

The statistical comparison revealed lower values of the maximal discharge frequency—the parameter “a” of the sigmoidal curve—in the Pseudo group in comparison to the CS + UCS mice but not the Naïve animals (Fig. 6A–C). However, other parameters of sigmoidal curves were similar between groups of mice (Fig. 6D and E). The analysis of the spike adaptation ratio showed that L4 VIP-AC exhibited a lower spiking adaptation index in the CS + UCS group than in the Naïve group but not in comparison to the Pseudo group (Fig. 6F).

Additional analysis of raw data and *F*–*I* curves confirmed reduced intrinsic excitability of VIP-AC in the Pseudo group in relation to the CS + UCS (Fig. S12). In contrast to sigmoidal curve analysis, the statistical comparison of *F*–*I* curves additionally revealed decreased intrinsic excitability in the Pseudo mice compared to the Naïve animals (Fig. S12).

The subsequent analysis of AP parameters in L4 VIP-AC revealed more depolarized threshold potential in the CS + UCS and Pseudo groups in relation to the Naïve mice (Fig. S13A and B) and lower AP amplitude in the Pseudo group compared to the Naïve animals (Fig. S13A and C). Moreover, the AP half-width was wider in the Pseudo group relative to the Naïve and CS + UCS mice (Fig. S13A and D), but no differences were found in fast AHP amplitude between groups (Fig. S13A and E). The above results suggest that conditioning and pseudoconditioning lead to changes in the AP shape of VIP-AC. The wider APs observed in the Pseudo group, as opposed to the Naïve and CS + UCS groups, may partly explain the diminished intrinsic excitability of VIP-AC in this group of mice.

Summarizing, statistical analysis of the sigmoidal and the *F*–*I* curves indicates that pseudoconditioning decreases intrinsic excitability of L4 VIP-AC.

### Learning does not influence intrinsic excitability of VIP-LTS

The second subtype of L4 VIP-INs that was analyzed consisted of cells with the LTS pattern. First, basic electrophysiological parameters of VIP-LTS from 3 groups of tested animals were analyzed. The measurements of resting potential and input resistance did not show any differences between groups of animals (Fig. S11D and E). However, a greater rheobase was found in the Pseudo group relative to the Naïve group (Fig. S11F).

**Fig. 7:**
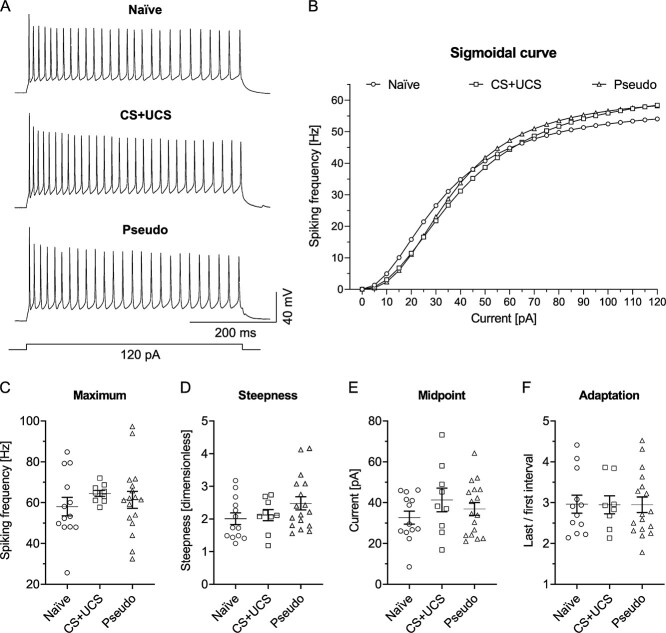
Conditioning and pseudoconditioning do not influence intrinsic excitability of L4 VIP-LTS. A) Examples of cell discharges and B) sigmoidal curves in tested groups of mice. C-F) No differences were observed in C) the curve’s maximum (Kruskal-Wallis test, *P* = 0.3091); D) the curve’s steepness (One-way ANOVA, F_(2, 36)_ = 1.800, *P* = 0.1799); E) the curve’s midpoint (One-way ANOVA, F_(2, 36)_ = 1.099, *P* = 0.3442); F) spike adaptation (One-way ANOVA, F_(2, 34)_ = 0.0016, *P* = 0.9984). B-E) Naïve = 13 (8), CS + UCS = 9 (8), Pseudo = 17 (17). F) Naïve = 12 (8), CS + UCS = 8 (7), Pseudo = 17 (17).

In the next step of VIP-LTS analysis, intrinsic excitability was examined using the sigmoidal curve fitting (Fig. 7).

The analysis of sigmoidal function parameters did not show any differences in the curve’s maximum, steepness, or midpoint values (Fig. 7B–E). Also, the analysis of spiking adaptation did not reveal any changes after CS + UCS or Pseudo (Fig. 7F).

We also performed an analysis of *F*–*I* curves for VIP-LTS, which showed differences between the Naïve and Pseudo groups (Fig. S14). The unexpected discrepancy between sigmoidal and *F*–*I* curve analyses may originate from the fact that more cells in the Pseudo group fire at higher current intensities than those from the Naïve group. Taking into account the similarities in the courses of the sigmoidal and the *F*–*I* curves between the Naïve and Pseudo groups, we can assume that VIP-LTS from both groups fire at the same frequency. However, cells from the Pseudo group can fire at higher currents, simultaneously presenting a similar maximal firing frequency as VIP-LTS from the Naïve group.

The AP analysis revealed more depolarized threshold potential in the Pseudo group compared to the Naïve mice (Fig. S15A and B). However, further analysis did not show any other differences in the AP properties (Fig. S15A, C–E).

In summary, CS + UCS or Pseudo does not affect the maximal firing frequency of L4 VIP-LTS. However, pseudoconditioning can enhance the capacity of VIP-LTS to discharge at higher frequencies.

## Discussion

Our present study demonstrates that intrinsic excitability of molecularly diverse interneurons in the neocortex changes specifically to the interneuron type and the form of learning. We found that conditioning leads to an increase in intrinsic excitability of L4 SST-LTS, whereas pseudoconditioning causes a decrease in intrinsic excitability of SST-LTS, PV-INs, and VIP-AC in L4 of the barrel cortex. Also, changes in intrinsic excitability were accompanied by changes in some features of an AP shape, such as spike threshold or spike half-width. In general, the increase in intrinsic excitability was accompanied by the narrowing of APs, whereas the decrease in intrinsic excitability was associated with the broadening of APs. In principle, changes in AP parameters such as spike threshold and duration or the amplitude of AHP have essential outcomes for intrinsic excitability. It has been found that inactivity of excitatory neurons drives a homeostatic increase in spike width (Li et al. 2020), while learning reduces the amplitude of AHP in hippocampal SST-INs (McKay et al. 2013), suggesting that changes in the AP parameters are important mechanisms of neuronal plasticity.

Interneuron-specific learning-evoked changes in intrinsic excitability can have a significant impact on the functioning of the L4 local circuit in the barrel cortex and can influence how this network processes and codes information. Therefore, changes in intrinsic excitability of GABAergic interneurons may have important roles in learning and memory consolidation in tasks based on the use of vibrissae.

In L4 of the barrel cortex, excitatory neurons and different interneuron types create a densely interconnected circuit network that is the main input for the axons from the ventrobasal complex of the thalamus (Chmielowska et al. 1989; Wimmer et al. 2010; Oberlaender et al. 2012; El-Boustani et al. 2020). Thalamocortical neurons innervate mainly excitatory neurons and PV-INs (Beierlein et al. 2003; Cruikshank et al. 2010; Sermet et al. 2019), whereas SST-INs and VIP-INs are much weaker excited by thalamocortical axons (Beierlein et al. 2003; Cruikshank et al. 2010; Sermet et al. 2019). Not only do L4 excitatory neurons create reciprocal connections with local PV-INs and SST-INs but also these interneurons are highly interconnected (Beierlein et al. 2003; Gabernet et al. 2005; Inoue and Imoto 2006; Ma et al. 2012; Xu et al. 2013; Koelbl et al. 2015; Scala et al. 2019). In general, PV-INs are responsible for the fast and strong feedforward inhibition, whereas SST-INs provide the delayed and weaker feedback inhibition (Beierlein et al. 2003; Gabernet et al. 2005; Inoue and Imoto 2006; Cruikshank et al. 2010; Ma et al. 2012; Feldmeyer et al. 2018). Interestingly, the activity of L4 SST-INs has a disinhibitory effect on the local network because these interneurons inhibit local PV-INs more effectively than excitatory neurons and hence reduce PV-IN-mediated inhibition of excitatory neurons and consequently enhance the activity of excitatory neurons (Ma et al. 2012; Xu et al. 2013). Therefore, changes in intrinsic excitability of SST-LTS and PV-INs may influence the complex activity of the local neural circuit and the way of information processing and coding. Research shows that cortical SST-INs regulate the sequential activity of pyramidal neurons arising from motor or visually guided active avoidance tasks (Makino and Komiyama 2015; Adler et al. 2019). Moreover, SST-INs and PV-INs of the barrel cortex promote the synchronization of spike times across cortical layers (Jang et al. 2020).

No detailed studies on synaptic inputs and outputs of L4 VIP-IN in the rodent somatosensory cortex have been published so far. However, considering that these cells often form disinhibitory circuits innervating SST-INs and PV-INs, it can be expected that this is also the case in L4 of the barrel cortex (Caputi et al. 2009; Lee et al. 2013; Pfeffer et al. 2013; Jiang et al. 2015; Walker et al. 2016; Kullander and Topolnik 2021). However, VIP-INs in the rodent cortex may also inhibit excitatory cells (Caputi et al. 2009; Pfeffer et al. 2013; Garcia-Junco-Clemente et al. 2017).

### Conditioning increases intrinsic excitability of low-threshold spiking SST-INs

A previous study based on the learning model used in our experiments has shown that conditioning is associated with an increased density of cells positive for SST and glutamate decarboxylase 67 (GAD67) in the barrels corresponding to the manipulated vibrissae (Cybulska-Klosowicz et al. 2013). This might indicate an increase in the SST-IN activity following the learning process. The latest research has shown that L4 SST-INs are essential in this conditioning paradigm (Dobrzanski et al. 2022). Now, we show that this simple model of associative learning leads to an increase in intrinsic excitability of L4 SST-INs characterized by the LTS pattern. Increased intrinsic excitability was accompanied by decreased amplitude and reduced half-width of APs. In contrast, conditioning does not affect intrinsic excitability of L4 PV-INs. Taking into account that L4 SST-INs are responsible for the disinhibitory effect (Xu et al. 2013), the increase in intrinsic excitability of SST-INs might be responsible for the higher activity of excitatory neurons after conditioning (Bekisz et al. 2010).

Studies using the trace eyeblink paradigm in mice have shown that conditioning leads to an increase in intrinsic excitability of hippocampal SST-INs, accompanied by a decrease in the amplitude of AHPs (McKay et al. 2013). Moreover, the results of other studies suggest that also the induction of LTP in the hippocampal CA1 SST-INs is accompanied by an increase in intrinsic excitability of these interneurons (Sammari et al. 2022). Furthermore, experiments on the mouse prefrontal cortex have shown an increase in the intrinsic excitability of SST-INs activated by fear conditioning (Cummings et al. 2022). Other experiments also suggest that the increased excitability of SST-INs in the mouse prefrontal cortex may take part in the morphine-induced conditioned place preference (Jiang et al. 2021). On the other hand, a decrease in intrinsic excitability of SST-INs has been observed in the paradigm of novel taste learning in mice (Gould et al. 2021). In this case, the decrease in intrinsic excitability of SST-INs in the anterior insular cortex was accompanied by an increase in the amplitude of medium AHPs (Gould et al. 2021). It has also been shown that an experimental reduction of the SST-IN excitability in the anterior insula positively affects the memory formation of a new taste (Gould et al. 2021).

In contrast, conditioning did not affect intrinsic excitability of L4 PV-INs in the barrel cortex. Previous studies have also not found any evidence of plastic changes in L4 PV-INs of the barrel cortex after conditioning (Siucinska and Kossut 2006; Tokarski et al. 2007; Bekisz et al. 2010). Furthermore, no changes have been observed in intrinsic excitability, resting potential, or input resistance of FS (presumably PV) interneurons, as well as no differences in the density of PV/GAD67 positive cells have been shown after CS + UCS (Siucinska and Kossut 2006; Tokarski et al. 2007; Bekisz et al. 2010).

### Pseudoconditioning decreases intrinsic excitability of SST-LTS, PV-INs, and VIP-AC

The analysis of raw data (but not sigmoidal fitting) showed that pseudoconditioning decreases intrinsic excitability of L4 SST-LTS (Fig. S4). However, this change was not accompanied by any alterations in basic electrophysiological parameters or AP shape differences compared with the Naïve group. For this reason, it is unclear whether changes in SST-LTS intrinsic excitability after pseudoconditioning have a functional role. In general, we may assume that weaker activity of SST-INs might lead to higher activity of excitatory neurons. However, SST-INs also inhibit PV-INs; thus, the net effect of weaker activity of L4 SST-INs might be a higher activity of PV-INs and thus stronger PV-IN-mediated inhibition of pyramidal cells (Ma et al. 2012; Xu et al. 2013).

Also, in the Pseudo group of animals, we observed a decrease in intrinsic excitability of L4 PV-INs accompanied by decreased input resistance, higher adaptation index, widening of an AP, and increased amplitude of fast AHP in these interneurons.

Finally, the analysis of learning-related effects on L4 VIP-IN intrinsic excitability suggests that a subset of these interneurons might undergo changes after pseudoconditioning. We observed reduced excitability of VIP-AC in the Pseudo group compared to the Naïve and CS + UCS groups (Fig. S12). Decreased intrinsic excitability of VIP-AC was accompanied by depolarized resting potential and threshold potential, lower spike amplitude, and broader spike half-width.

The effects of learning paradigms are not consistent and conclusive in terms of VIP-LTS.

Decreased intrinsic excitability of almost all interneuron types might be a common mechanism of a certain form of nonassociative learning after pseudoconditioning or a kind of habituation to irrelevant information—repetitive sensory stimulation of vibrissae. It has been shown that both PV-INs and SST-INs in the primary auditory cortex adapt relatively quickly to repeated sounds and regulate the activity of excitatory cells in response to both rare and frequent stimuli (Natan et al. 2015). Studies on mice lacking the GluN1 subunit of the NMDA receptor in PV-INs have shown that these animals exhibit impairment in habituation, working memory, and associative learning (Carlén et al. 2012). Also, in L4 of the barrel cortex, the adaptation of the neuronal activity in response to a high-frequency vibrissal deflection (sensory adaptation) occurs in both excitatory cells and GABAergic neurons, most likely PV-INs (Khatri and Simons 2006). Interestingly, the rapid sensory adaptation to repetitive vibrissal stimulation depends on the brain state, being more prominent in the resting state (Castro-Alamancos 2004). However, as animals learn to perform tasks efficiently, sensory adaptation exhibits the same strong level as during the states of low arousal (Castro-Alamancos 2004). Other experiments involving prolonged stimulation of vibrissae have shown that the adaptation is not accompanied by changes in resting potential, input resistance, or intrinsic excitability of neurons in the barrel cortex—the majority of these cells were excitatory and originated from layers 2 to 4 (Chung et al. 2002). Therefore, it is unclear whether rapid sensory adaptation has an impact on the intrinsic parameters of PV-INs.

Further studies are required to understand the role of decrease in intrinsic excitability of PV-INs after pseudoconditioning. It is unclear whether this is a form of long-lasting habituation to a repetitive and noninformative stimulus or if this is a more complex process of nonassociative learning. Previous works have shown a significantly higher density of excitatory synapses in the barrels corresponding to the manipulated vibrissae in Pseudo animals as well as in mice receiving the CS stimulus only compared to the Naïve and CS + UCS mice (Jasinska et al. 2010). Furthermore, it has been shown that there are specific changes exclusively associated with Pseudo, manifested as an increase in the density of dendritic spines in the barrels corresponding to manipulated vibrissae (Jasinska et al. 2010). Later studies have revealed that pseudoconditioning leads to a global decrease in the density of cannabinoid receptor 1 (CB1) immunopositive puncta in the barrels of all rows on both sides of the snout corresponding to the manipulated as well as nonmanipulated vibrissae (Siucinska et al. 2018). The decrease in the number of CB1 has been characteristic only for the pseudoconditioned group and has not been observed in the CS + UCS, Naïve, CS-only, or UCS-only groups. Based on these studies, pseudoconditioning can be recognized as a more complex form of nonassociative learning. Presumably, the decrease in the excitability of PV-INs, caused by pseudoconditioning, can lead to weaker inhibition of L4 excitatory cells and an overall increase in the network activity, as indicated by the increased density of dendritic spines and excitatory synapses (Jasinska et al. 2010). Additionally, the reduction in the number of CB1 in excitatory neurons might lead to an increase in the excitability of these cells (Domenici et al. 2006). Perhaps pseudoconditioning leads to a nonspecific and generalized process as opposed to associative learning. In this sense, reduced excitability of PV-INs can lead to not only increased activity of excitatory neurons but also the reduction in the accuracy of information transfer by lowering the precision of excitatory cell discharges. This, in turn, can lead to long-term depression of excitatory transmission (Celikel et al. 2004). In general, decreased intrinsic excitability of PV-INs can disturb the balance between the excitation and inhibition in the local network and lead to the weakening of neuronal functions related to learning (Gandal et al. 2012; Campanac et al. 2013; Toader et al. 2020).

L4 VIP-AC of the barrel cortex may be involved, similarly to local PV-INs, in a mechanism opposed to conditional learning. It has been found that in the basolateral amygdala nucleus (BLA), VIP-INs respond strongly to an unconditioned electrical stimulus, and their activity decreases during learning as the animal gains experience (Krabbe et al. 2019). Interestingly, the activity of BLA VIP-INs can again be increased by using an electrical stimulus that is not paired with a CS or by applying an UCS with the increased strength. These results suggest that BLA VIP-INs are activated by new and unexpected situations (Krabbe et al. 2019), which imposes the requirement on the animal to revise previous experiences and expectations. VIP-INs of BLA could be involved in the process of the differentiation between important and irrelevant stimuli. We may hypothesize that L4 VIP-AC can also participate in such a mechanism in the barrel cortex.

## Conclusions

Presented experiments show that changes in intrinsic excitability of GABAergic interneuron subtypes are universal mechanisms of learning. Alterations in intrinsic excitability of GABAergic interneurons may influence many mechanisms related to information processing. Changes in intrinsic excitability may lead to a modification of the process of spatial and/or temporal summation of synaptic inputs (Magee 1999; Wang et al. 2003; Lee and Kwag 2012)**.** Temporal synaptic summation is directly related to the neuronal ability to filter incoming information and may exhibit characteristics similar to those of a high-pass filter, which blocks low-frequency signals (Engbers et al. 2012). The mechanism of signal filtering is important in the way how neurons and networks process information. In addition, modifications of the synaptic summation can lead to the phenomenon known as EPSP to spike (E-S) potentiation (Bliss and Lømo 1973; Daoudal et al. 2002; Daoudal and Debanne 2003). The E-S potentiation entails the increased sensitivity to an input signal and, in this way, enhances the dynamic range of the response of an individual neuron to incoming stimulation (Pouille et al. 2009). Overall, intrinsic excitability, along with related temporal synaptic summation and increased sensitivity to incoming signals, can regulate AP reliability, thereby influencing information accuracy (Magee 1999; Sourdet et al. 2003; Pang and Fairhall 2019). Increased sensitivity to input signal may also result in a higher precision of the spike timing (Sourdet et al. 2003; Losonczy et al. 2008; Mahon and Charpier 2012).

Summarizing, the presented results add essential knowledge to our understanding of the role of molecularly distinct GABAergic interneurons in the neocortex during the learning process. However, it is not known precisely how changes in intrinsic excitability of GABAergic interneurons affect information processing and memory formation in the cortex. Further complex experiments are necessary to understand how changes in intrinsic excitability of specific subpopulations of GABAergic interneurons shape the information processing and coding along with animal performance.

## Supplementary Material

Supplement_Kanigowski_and_Urban-Ciecko_bhae109
