# Supplementary material for: Conditioning and pseudoconditioning differently change intrinsic excitability of inhibitory interneurons in the neocortex
Source: Cereb Cortex. 2024 Apr 3;34(4):bhae109. doi: 10.1093/cercor/bhae109 (PMC10993172; doi:10.1093/cercor/bhae109)
Supplement: Supplement_Kanigowski_and_Urban-Ciecko_bhae109 [file supplement_kanigowski_and_urban-ciecko_bhae109.pdf]

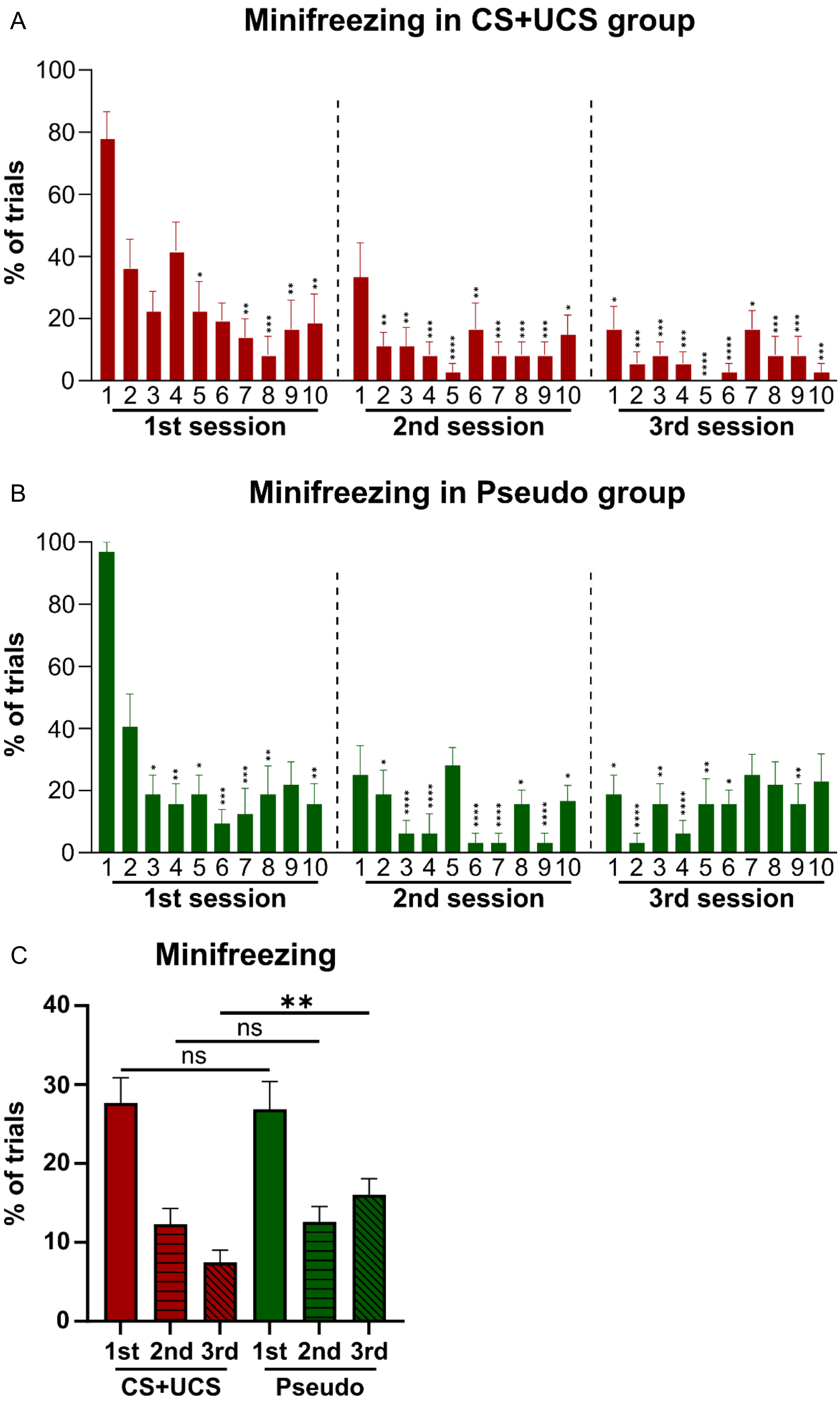
A
Minifreezing in CS+UCS group
% of trials
100
80
60
40
20
0
1 2 3 4 5 6 7 8 9 10
1st session
2nd session
3rd session
B
Minifreezing in Pseudo group
% of trials
1 2 3 4 5 6 7 8 9 10
1st session
2nd session
3rd session
C
Minifreezing
% of trials
40
30
20
10
0
**
ns
ns
1st 2nd 3rd 1st 2nd 3rd
CS+UCS
Pseudo

Fig. S1. Behavioral assessment of learning in the CS+UCS and Pseudo groups of SST-Ai14 mice.

Decrease in the percentage of head movements (minifreezing) in consecutive minutes of **(A)** conditioning and **(B)** pseudoconditioning. Asterisks present statistical differences between the first minute of the first session and all other minutes in the training (Kruskal-Wallis test followed by Dunn's test, $p<0.05$). **(C)** The CS+UCS group of mice presents a higher degree of freezing (lower percentage of head turns) on the third day of training compared to the Pseudo group on the same day (Kruskal-Wallis test, $p<0.0001$; Dunn's test, $p=0.0064$).

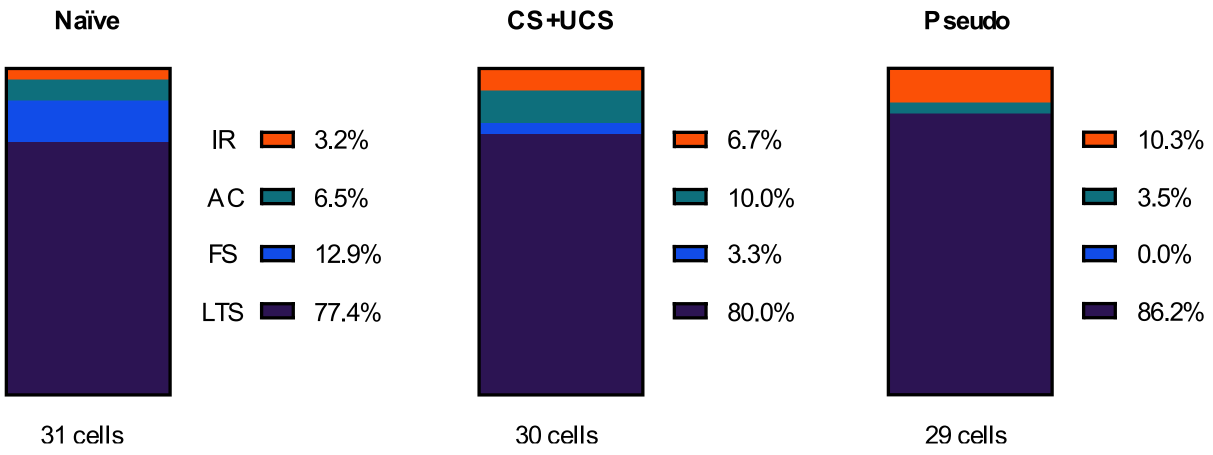

Fig. S2. Population distribution of electrophysiological subtypes of L4 SST-INs in three groups of mice.

The statistical analysis did not reveal changes in the fraction of SST-LTS between CS+UCS (2-sample test for equality of proportions with continuity correction, X^2=6.8949^{-31}, p=1) or Pseudo (X^2=0.2973, p=0.5856) compared to the Naïve group. No changes were observed in the fraction of SST-FS between CS+UCS (X^2=0.80168, p=0.3706) or Pseudo (X^2=2.2036, p=0.1377) compared to the Naïve mice. No changes in the fraction of SST-AC between CS+UCS (X^2=0.0014641, p=0.9695) or Pseudo (X^2=3.2972^{-31}, p=1) compared to the Naïve animals. No changes in the fraction of SST-IR between CS+UCS (X^2=0.00084816, p=0.9768) or Pseudo (X^2=0.34443, p=0.5573) compared to the Naïve group. Naïve=31(16), CS+UCS=30(13), Pseudo=29(8).

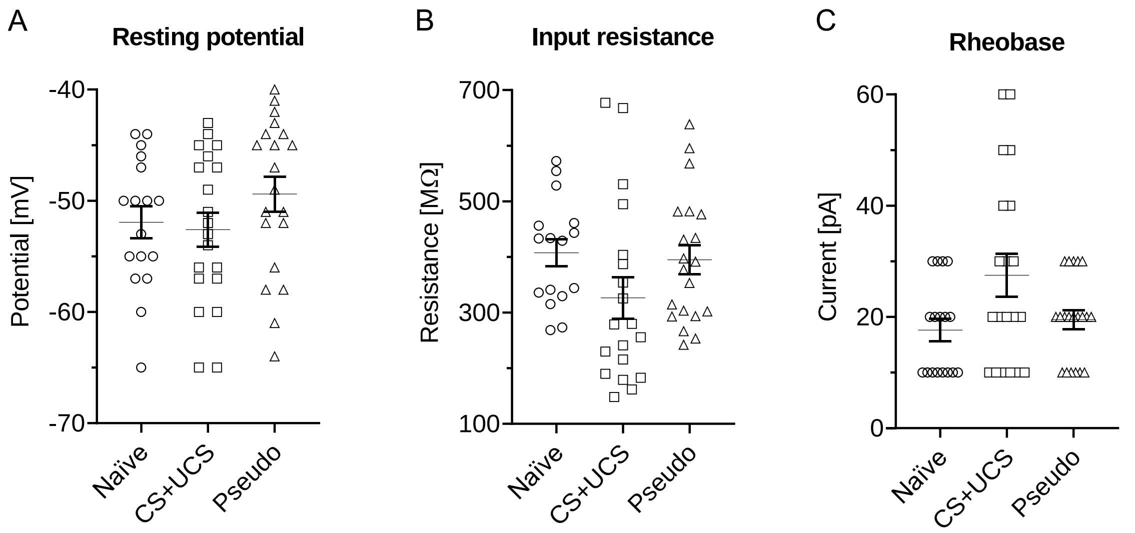

Fig. S3. Basic electrophysiological properties of L4 SST-LTS in three groups of mice. No changes were observed in: **(A)** resting membrane potential (One-way ANOVA, $F_{(2, 54)}=1.279$, $p=0.2866$); **(B)** input resistance (One-way ANOVA, $F_{(2, 52)}=2.061$, $p=0.1376$); **(C)** rheobase (Kruskal-Wallis test, $p=0.1899$). **(A, C)** Naïve=17(7), CS+UCS=20(10), Pseudo=20(6). **(B)** Naïve=16(7), CS+UCS=19(10), Pseudo=20(6).

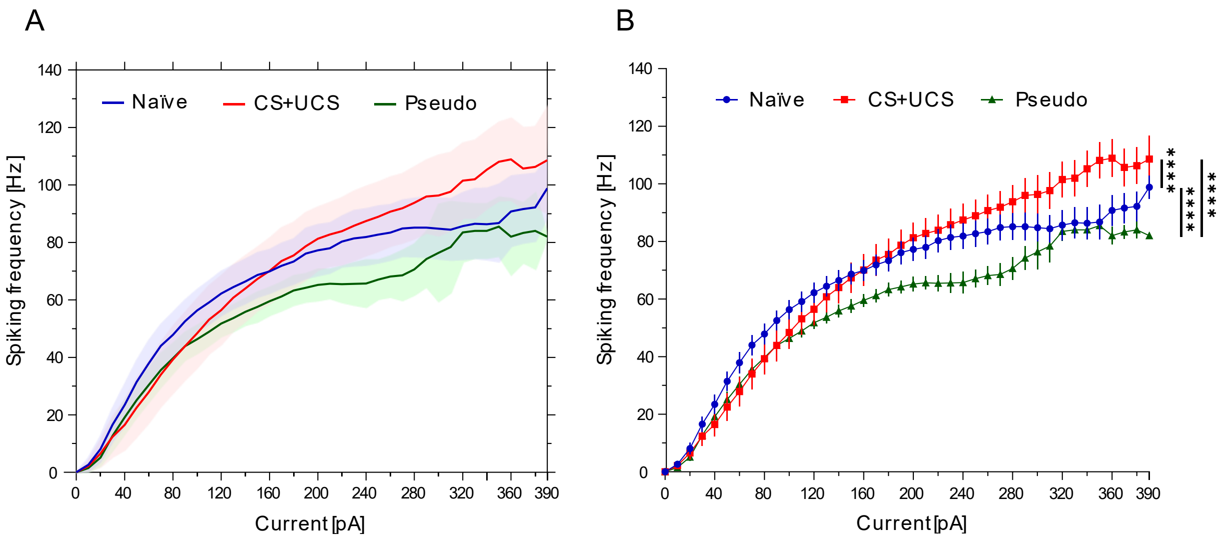

Fig. S4. F-I curves of SST-LTS intrinsic excitability.

(A) Curves depicting the mean intrinsic excitability, along with 95% confidence intervals, are presented for three groups of tested mice. **(B)** Same as A but means with SEM. Kolmogorov-Smirnov test showed changes between the Naïve and CS+UCS groups (p<0.0001), between the CS+UCS and Pseudo groups (p<0.0001), and also between the Naïve and Pseudo groups of mice (p<0.0001). Naïve=18(8), CS+UCS=21(10), Pseudo=20(6). The analysis incorporates cells that were excluded from sigmoidal curve analysis because of parameter "c" values that exceeded three standard deviations (n=1 in Naïve and n=1 in CS+UCS).

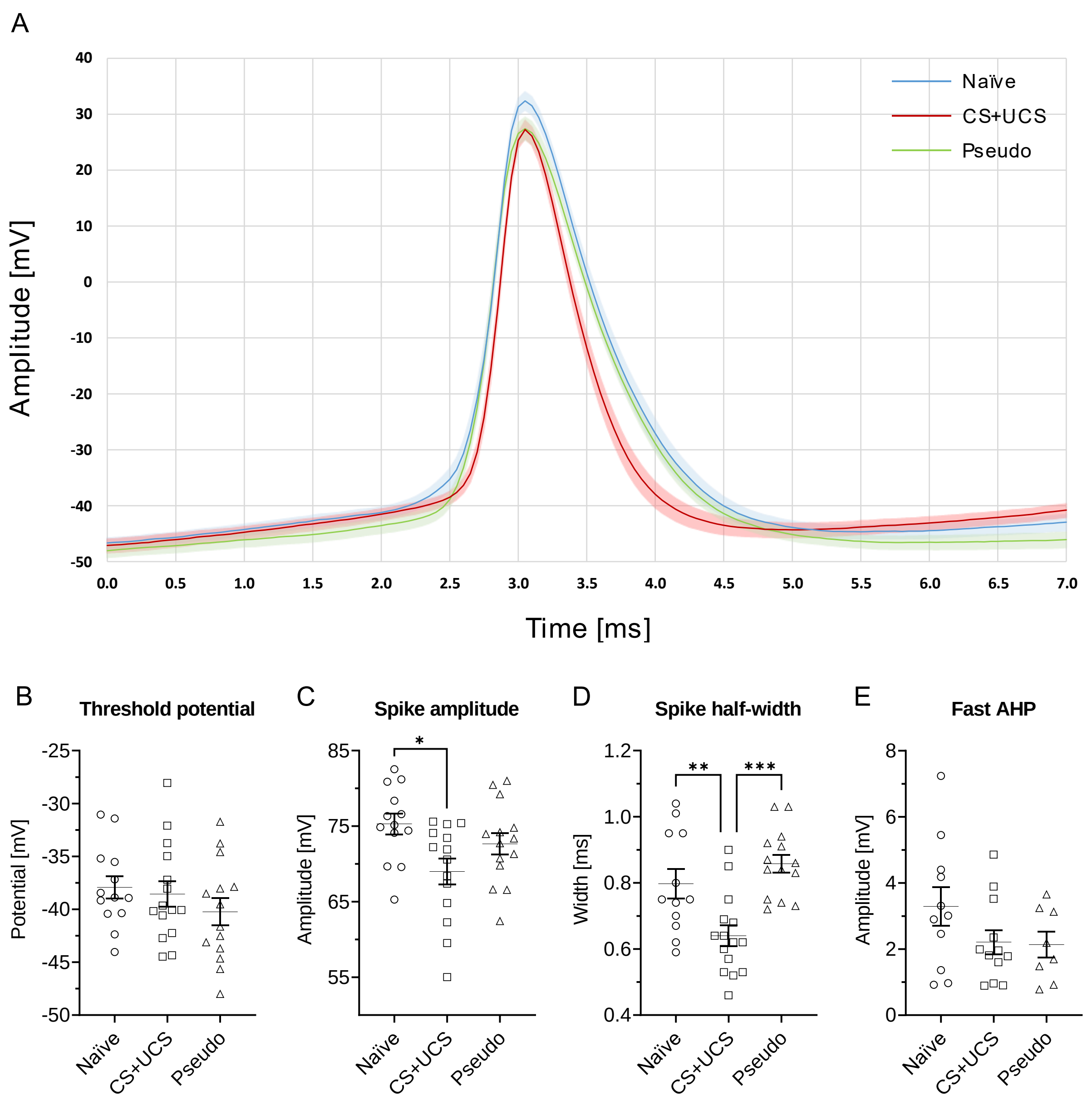

Fig. S5. Conditioning influences AP parameters of L4 SST-LTS.

(A) Averaged AP waveforms were presented as solid lines, while colored shadows represent SEM. Naïve=13(5), CS+UCS=15(10), Pseudo=14(6). **(B)** Threshold potential did not differ between groups (One-way ANOVA, $F_{(2, 39)}$=0.9847, p=0.3826); Naïve=13(5), CS+UCS=15(10), Pseudo=14(6). **(C)** AP amplitude was lower in the CS+UCS mice compared to the Naïve group (One-way ANOVA, $F_{(2, 38)}$=4.253, p=0.0215; Tukey's test, p=0.0167); Naïve=13(5), CS+UCS=14(10), Pseudo=14(6). **(D)** AP half-width in the CS+UCS group was narrower compared to the Naïve group (One way ANOVA, $F_{(2, 38)}$=11.62, p=0.0001; Tukey's test, p=0.0069) and Pseudo group (Tukey's test, p=0.0001); Naïve=12(5), CS+UCS=15(10),

Pseudo=14(6). **(E)** No differences were observed in fast AHP amplitude between groups of mice (One way ANOVA, $F_{(2, 28)}=1.929$, $p=0.1641$); Naïve=11(4), CS+UCS=12(10), Pseudo=8(5).

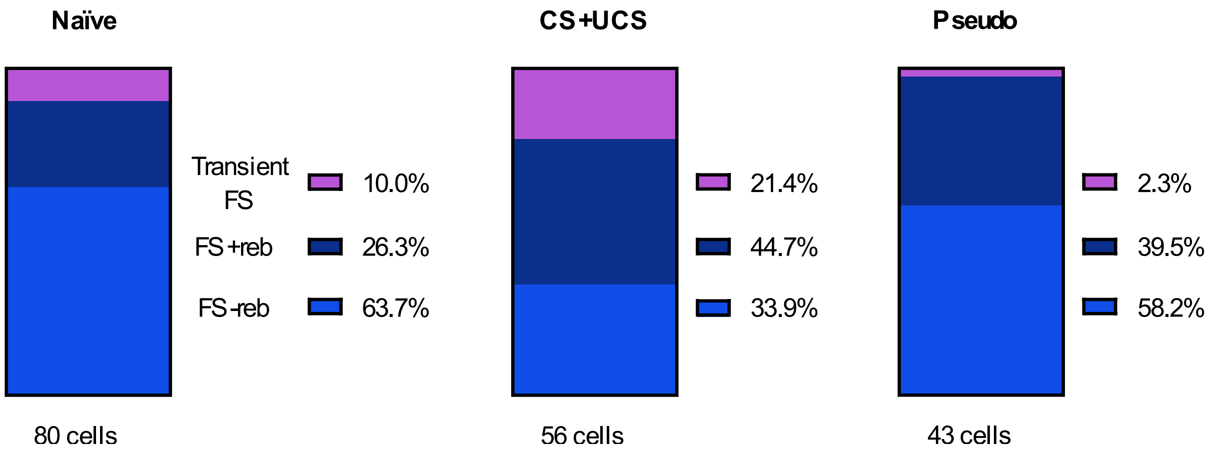

Fig. S6. Population distribution of electrophysiological subtypes of L4 PV-INs in three groups of mice.

The statistical analysis revealed a lower fraction of FS -reb. in the CS+UCS group compared to the Naïve group (2-sample test for equality of proportions with continuity correction, X^2=10.565, p=0.001153). However, no differences were observed in the fraction of FS +reb. between the Naïve and Pseudo groups (X^2=0.1731, p=0.6774). The analysis also showed a higher percentage of FS +reb. in the CS+UCS mice relative to the Naïve group (X^2=4.1909, p=0.04064). However, no difference was observed in the fraction of FS +reb. between the Naïve and Pseudo groups (X^2=1.7316, p=0.1882). The analysis of the fraction of Transient FS in cell populations did not show any differences between the Naïve and CS+UCS mice (X^2=2.5795, p=0.1083) or the Naïve and Pseudo mice (X^2=1.4291, p=0.2319).

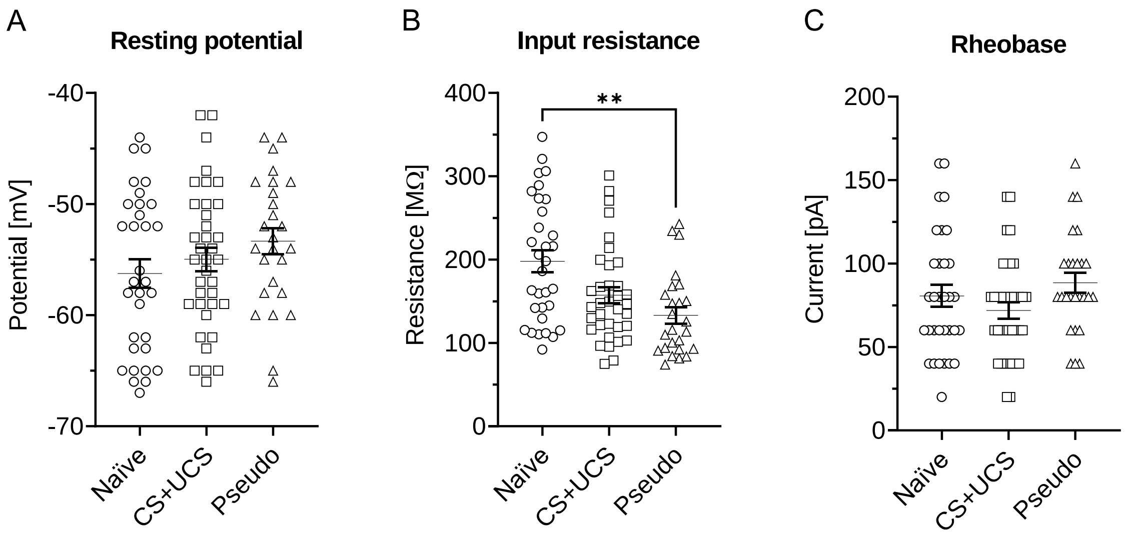

Fig. S7. Basic electrophysiological properties of L4 PV-INs in three groups of mice.

(A) No changes in resting membrane potential were shown between groups of mice (Kruskal-Wallis test, p=0.2991). **(B)** PV-INs in the Pseudo group showed reduced input resistance compared to cells of the Naïve group (Kruskal-Wallis test, p=0.0017; Dunn's test, p=0.0012). **(C)** The mean rheobase values did not differ between groups (One-way ANOVA, $F_{(2, 92)}$=2.010, p=0.1398). **(A, C)** Naïve=32(14), CS+UCS=37(12), Pseudo=26(10). **(B)** Naïve=32(14), CS+UCS=35(12), Pseudo=25(10).

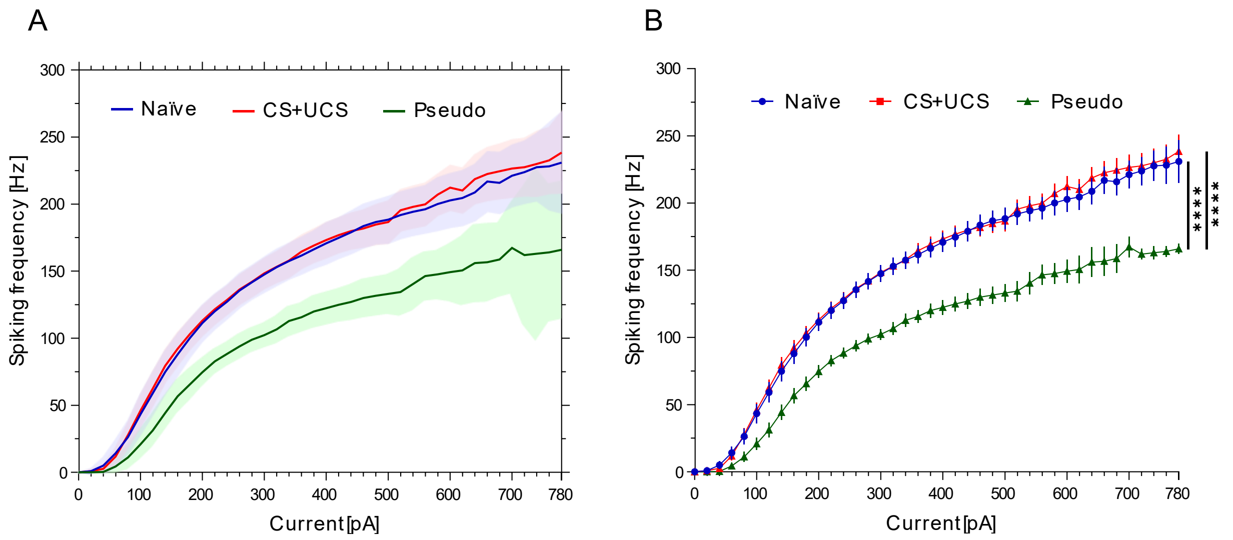

Fig. S8. F-I curves of PV-IN intrinsic excitability.

(A) Curves depicting the mean intrinsic excitability, along with 95% confidence intervals, are presented for three groups of tested mice. **(B)** Same as A but means with SEM. Statistical comparison revealed differences between the Naïve and Pseudo groups (Kolmogorov-Smirnov test, p<0.0001), and also between CS+UCS and Pseudo groups (p<0.0001). No difference was observed between the Naïve and CS+UCS mice (p=0.1257). Naïve=32(14), CS+UCS=37(12), Pseudo=26(10). The analysis incorporates cells that were excluded from sigmoidal curve analysis because of parameter "c" values that exceeded three standard deviations (n=3 in CS+UCS and n=1 in Pseudo).

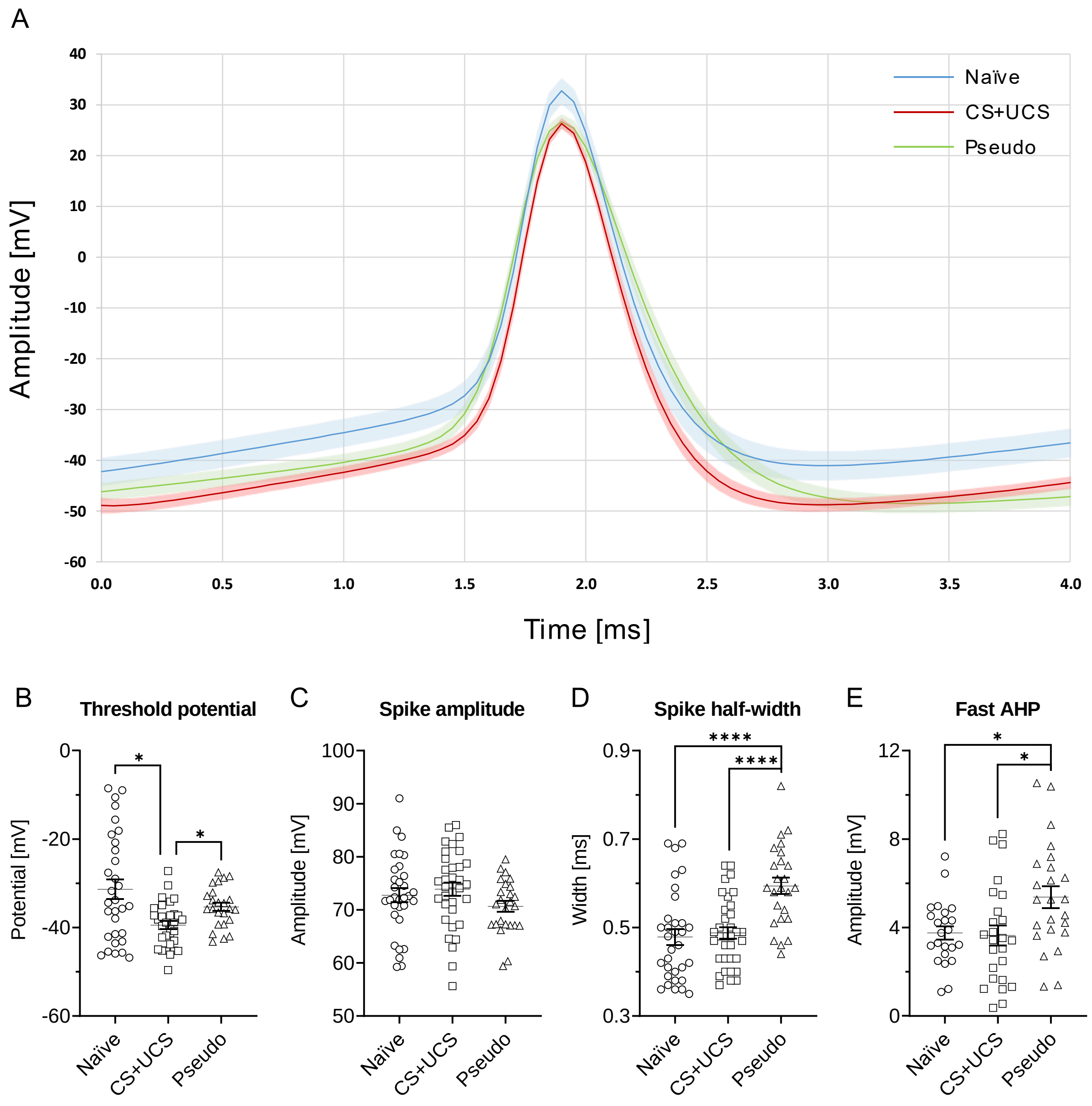

Fig. S9. Conditioning and pseudoconditioning influence AP parameters of PV-INs.

(A) Averaged AP waveforms were presented as solid lines, while colored shadows represent SEM. **(B)** The threshold potential of APs was hyperpolarized in the CS+UCS group compared to the Naïve (Kruskal-Wallis test, p=0.0083; Dunn's test, p=0.0137) and Pseudo groups (Dunn's test, p=0.0457). **(C)** No differences in AP amplitude between groups (One-way ANOVA, $F_{(2, 88)}$=1.638, p=0.2003). **(D)** In the Pseudo group, the AP half-width was wider relative to the AP half-width of the Naïve mice (One-way ANOVA, $F_{(2, 88)}$=13.49, p<0.0001; Tukey's test, p<0.0001) and CS+UCS groups (Tukey's test, p<0.0001). **(E)** The amplitude of fast AHPs was higher in the Pseudo group in comparison to the Naïve (One-way ANOVA, $F_{(2, 69)}$=5.164,

p=0.0081; Tukey's test, p=0.0269) and CS+UCS groups (Tukey's test, p=0.0136). **(A, C, D)** Naïve=32(14), CS+UCS=34(11), Pseudo=25(10). **(B)** Naïve=31(14), CS+UCS=31(11), Pseudo=24(10). **(E)** Naïve=23(13), CS+UCS=25(10), Pseudo=24(10).

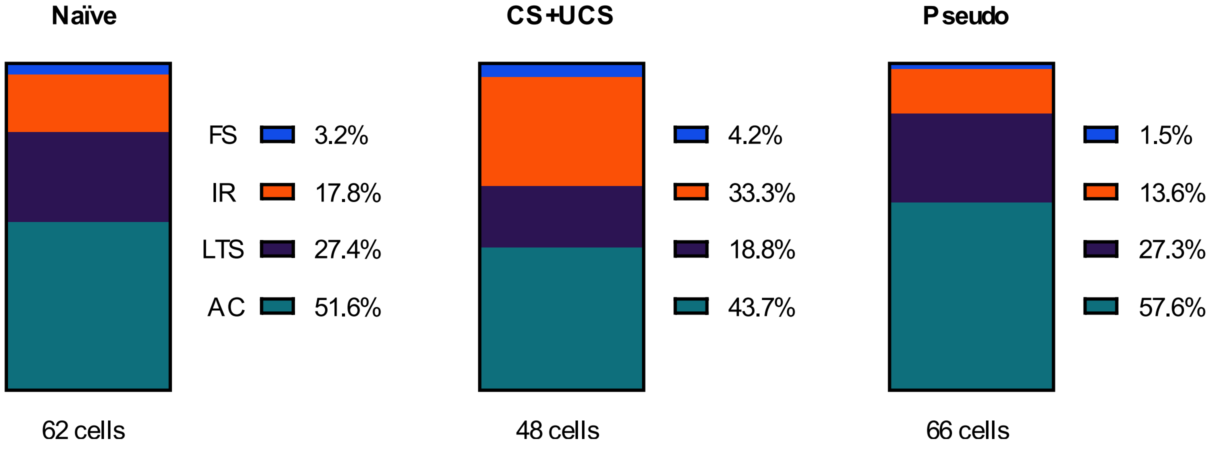

Fig. S10. Population distribution of electrophysiological subtypes of L4 VIP-INs in three groups of mice.

The statistical analysis did not show any difference in the fraction of AC cells between CS+UCS (2-sample test for equality of proportions with continuity correction, X^2=0.39203, p=0.5312) or Pseudo (X^2=0.24963, p=0.6173) compared to the Naïve group. No differences were observed in the fraction of LTS cells between CS+UCS (X^2=0.69743, p=0.4036) or Pseudo (X^2=2.7291^{-34}, p=1) compared to the Naïve mice. Also, no changes were observed in the fraction of IR cells between CS+UCS (X^2=2.7591, p=0.0967) or Pseudo (X^2=0.15663, p=0.6923) in relation to the Naïve group. The statistical comparison also did not reveal any difference in the fraction of FS cells between CS+UCS (X^2=5.2774^{-31}, p=1) or Pseudo (X^2=0.0030029, p=0.9563) compared to the Naïve animals.

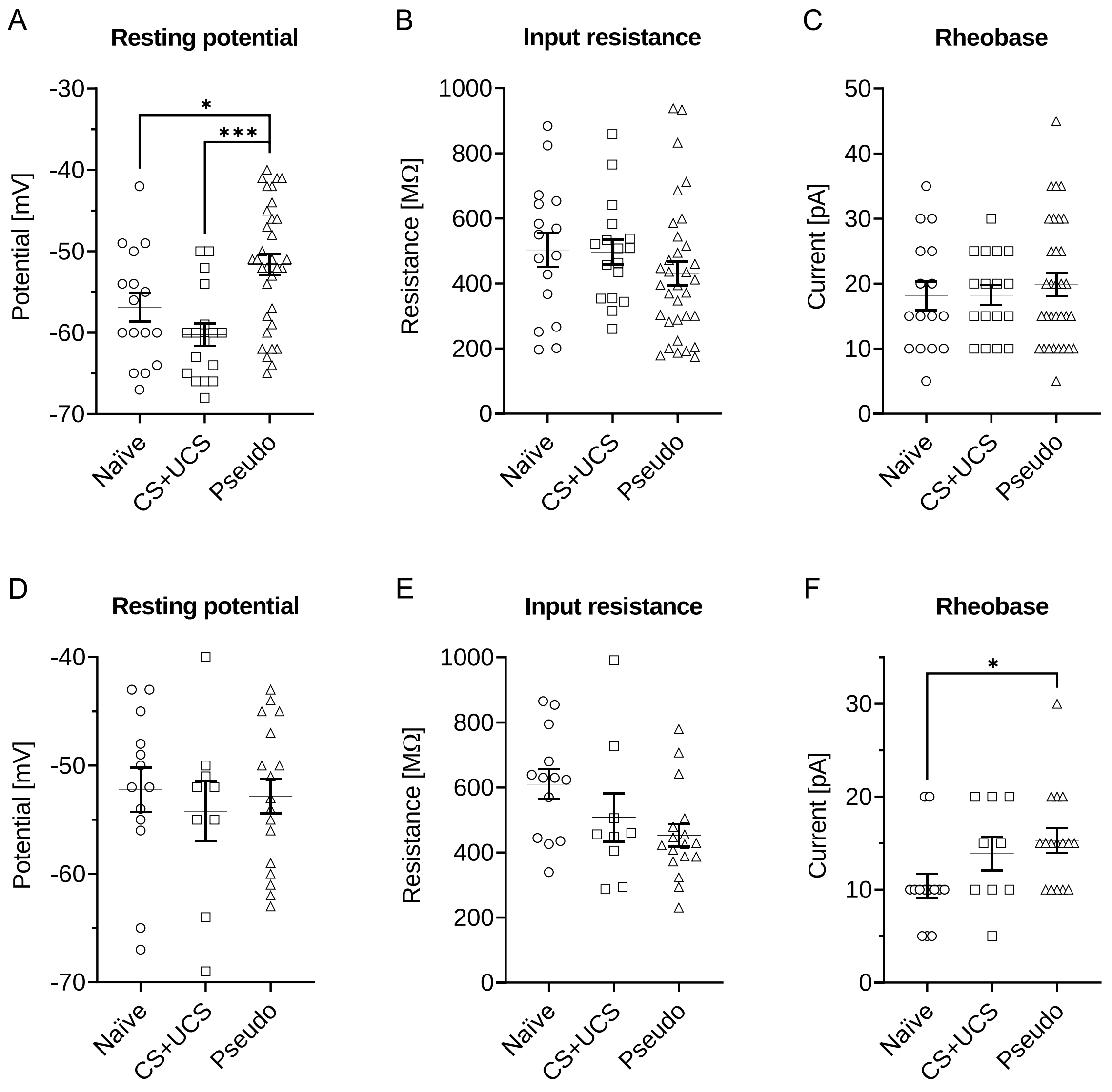

Fig. S11. Basic electrophysiological properties of L4 VIP-INs in three groups of mice. (A-C) Properties of VIP-AC: **(A)** The resting potential was depolarized in the Pseudo group in relation to the Naïve (One-way ANOVA, $F_{(2, 63)}$=9.060, p=0.0003; Tukey's test, p=0.0447) and CS+UCS mice (Tukey's test, p=0.0004); **(B)** No differences in input resistance (One-way ANOVA, $F_{(2, 63)}$=1.013, p=0.3689); **(C)** No changes in rheobase (One-way ANOVA, $F_{(2, 62)}$=0.2926, p=0.7474). **(D-F)** Properties of VIP-LTS: **(D)** No differences in membrane resting potential between groups of mice (One-way ANOVA, $F_{(2, 36)}$=0.2013, p=0.8186); **(E)** No differences in input resistance (Kruskal-Wallis test, p=0.0573); **(F)** Higher rheobase in the Pseudo group compared to the Naïve group (Kruskal-Wallis test, p=0.0329; Dunn's test, p=0.0317). **(A, B)** Naïve=16(11), CS+UCS=17(10), Pseudo=33(20). **(C)** Naïve=16(11),

CS+UCS=17(10), Pseudo=32(19). **(D, E)** Naïve=13(8), CS+UCS=9(8), Pseudo=17(17). **(F)** Naïve=13(8), CS+UCS=9(8), Pseudo=16(16).

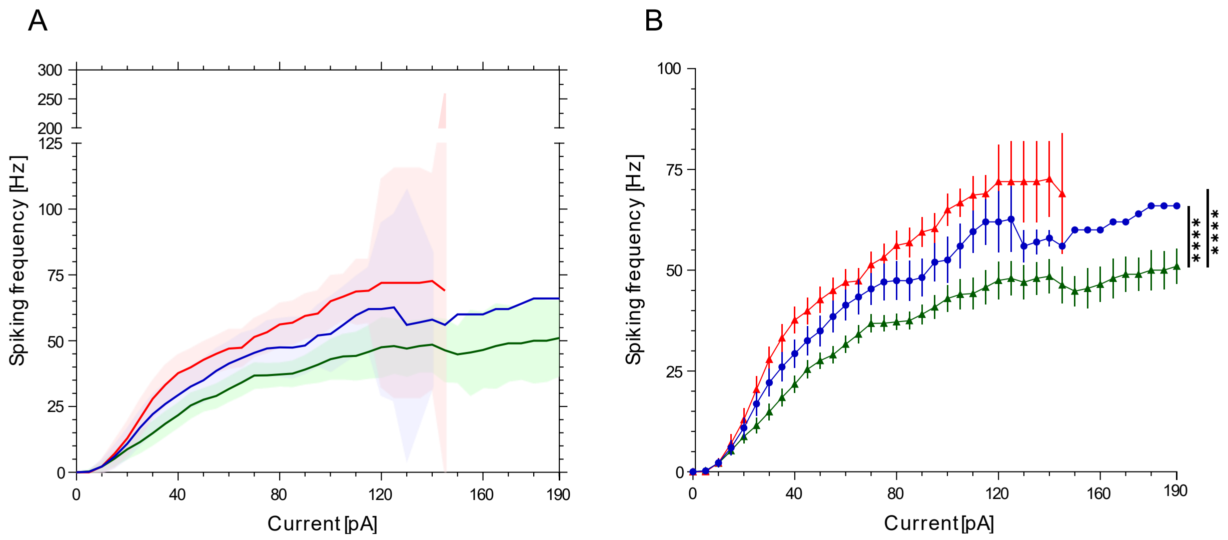

Fig. S12. F-I curves of VIP-AC intrinsic excitability.

(A) Curves depicting the mean intrinsic excitability, along with 95% confidence intervals, are presented for three groups of tested mice. **(B)** Same as A but means with SEM. Kolmogorov-Smirnov test revealed changes between the Naïve and Pseudo groups (p<0.0001), and also between CS+UCS and Pseudo groups (p<0.0001). No difference between the Naïve and CS+UCS groups was observed (p=0.0627). Naïve=18(13), CS+UCS=19(12), Pseudo=38(23). The analysis incorporates cells that were excluded from sigmoidal curve analysis because of parameter “c” that exceeded three standard deviations (n=3 in Naïve, n=2 in CS+UCS and n=5 in Pseudo).

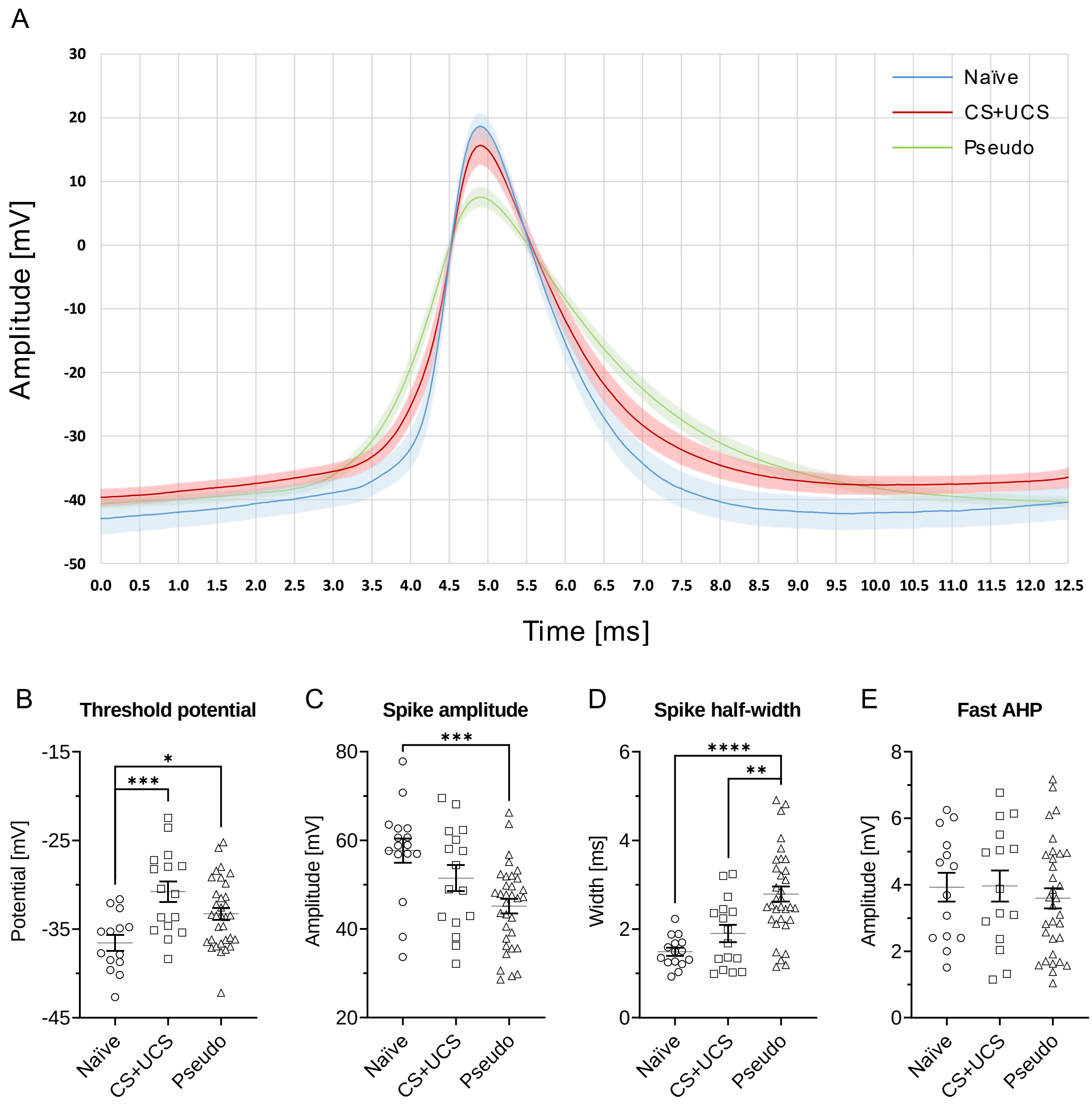

Fig. S13. Conditioning and pseudoconditioning influence AP parameters of VIP-AC.

(A) Averaged AP waveforms were presented as solid lines, while colored shadows represent SEM. Naïve=16(11), CS+UCS=16(9), Pseudo=33(20). **(B)** The threshold potential was depolarized in the CS+UCS (One-way ANOVA, $F_{(2, 60)}$=7.983, p=0.0008; Tukey's test, p=0.0005) and Pseudo groups (Tukey's test, p=0.0303) compared to the Naïve group; Naïve=14(9), CS+UCS=16(9), Pseudo=33(20). **(C)** The AP amplitude in the Pseudo group was lower in contrast to the amplitude of the Naïve group (One-way ANOVA, $F_{(2, 62)}$=8.167, p=0.0007; Tukey's test, p=0.0005); Naïve=16(11), CS+UCS=16(9), Pseudo=33(20). **(D)** The APs in the Pseudo group were wider relative to the APs in the Naïve (Kruskal-Wallis test,

p<0.0001; Dunn’s test, p=<0.0001) and CS+UCS groups (Dunn’s test, p=0.0100); Naïve=15(10), CS+UCS=16(9), Pseudo=33(20). **(E)** No differences in fast AHP amplitudes between groups (One-way ANOVA, $F_{(2, 58)}$=0.3174, p=0.7293); Naïve=14(10), CS+UCS=15(8), Pseudo=32(19).

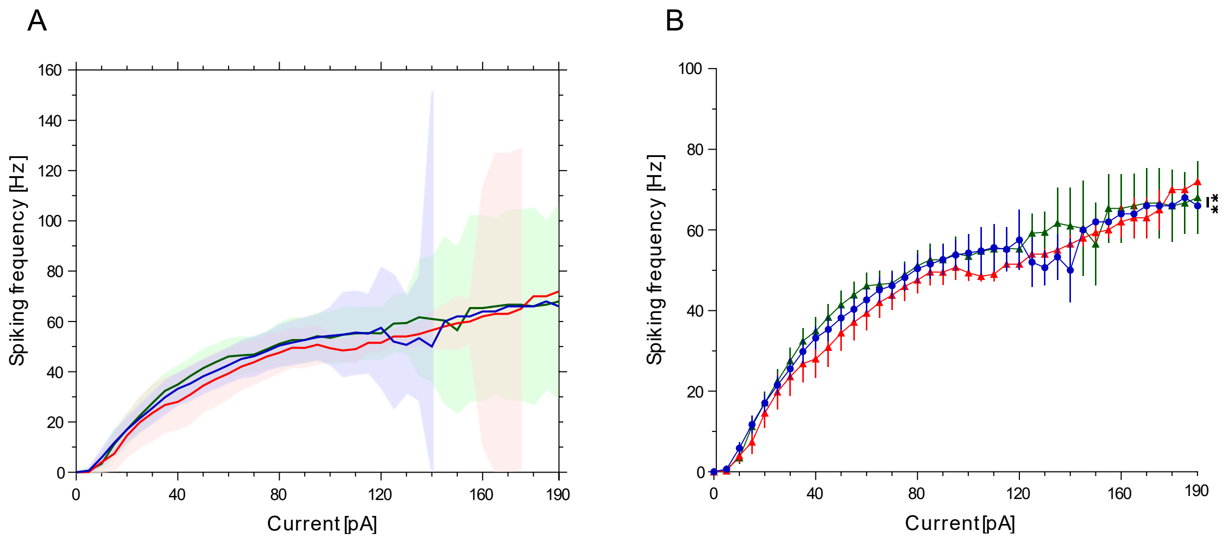

Fig. S14. F-I curves of VIP-LTS intrinsic excitability.

(A) Curves depicting the mean intrinsic excitability, along with 95% confidence intervals, are presented for three groups of tested mice. **(B)** Same as A but means with SEM. Kolmogorov-Smirnov test showed a difference between the Naïve and Pseudo groups (p=0.0087). No differences were observed between the Naïve and CS+UCS groups (p=0.2196) or between CS+UCS and Pseudo groups (p=0.5154). Naïve=15(10), CS+UCS=10(9), Pseudo=18(18). The analysis incorporates cells that were excluded from sigmoidal curve analysis because of values of parameters “a”, “b” or “c” that exceeded three standard deviations (n=1 in Naïve, n=1 in CS+UCS) or F-I curve was poorly fitted to the model (n=1 in Pseudo).

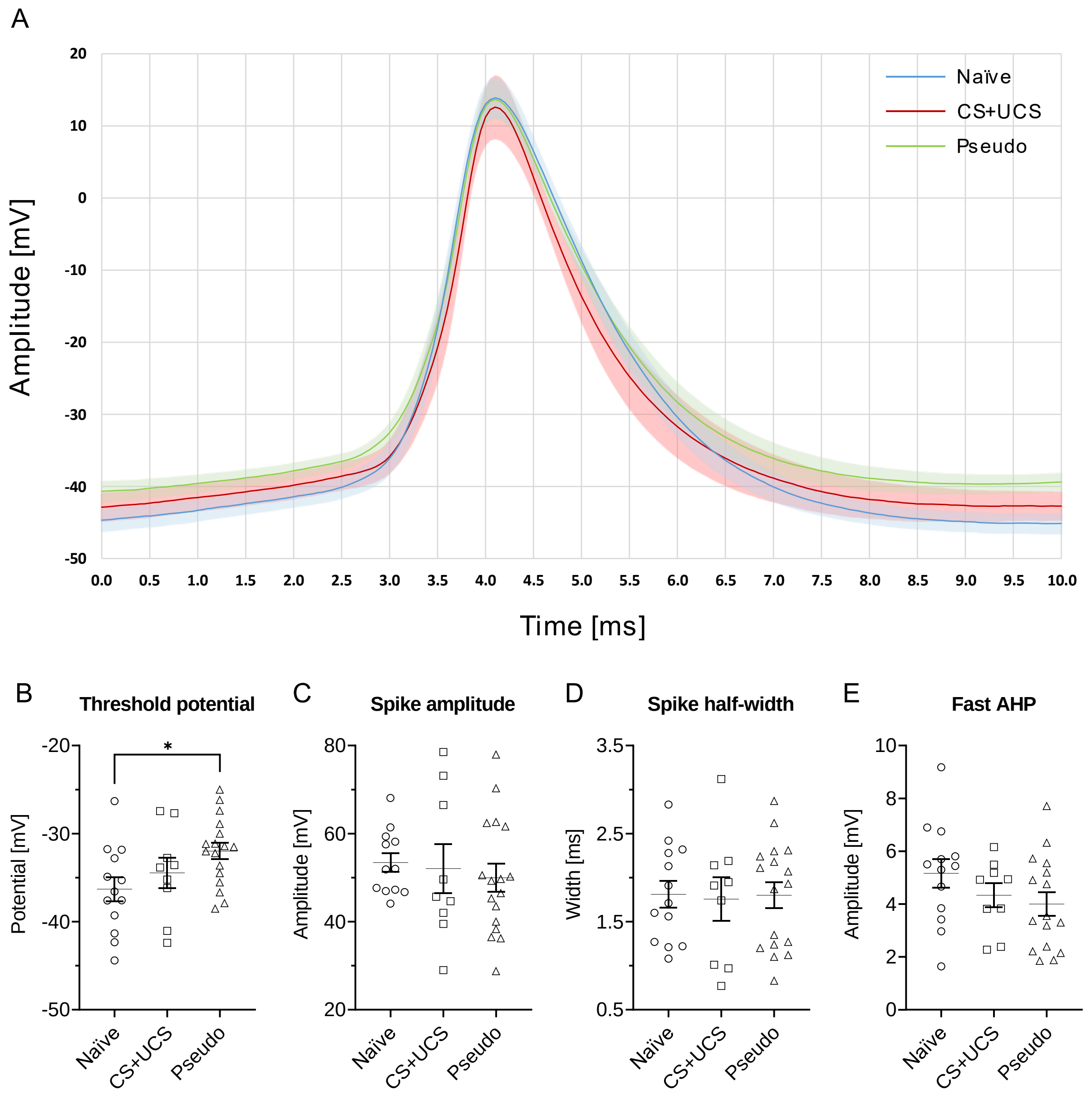

Fig. S15. Conditioning and pseudoconditioning do not influence most AP parameters in VIP-LTS.

(A) Averaged AP waveforms were presented as solid lines, while colored shadows represent SEM. **(B)** The threshold of APs was depolarized in the Pseudo group compared to the Naïve group (One-way ANOVA, $F_{(2, 36)}$=3.391, p=0.0447; Tukey's test, p=0.0366). **(C-E)** No changes were observed in **(C)** AP amplitude (One-way ANOVA, $F_{(2, 35)}$=0.2687, p=0.7659); **(D)** AP half-width (One-way ANOVA, $F_{(2, 36)}$=0.02280, p=0.9775); **(E)** fast AHP amplitude (One-way ANOVA, $F_{(2, 35)}$=1.621, p=0.2122). **(A, B, D)** Naïve=13(8), CS+UCS=9(8), Pseudo=17(17).

(C) Naïve=12(8), CS+UCS=9(8), Pseudo=17(17). **(E)** Naïve=13(8), CS+UCS=9(8), Pseudo=16(16).
